# PP2A Activation as a Senotherapeutic in Neuroblastoma

**DOI:** 10.3390/cancers18193088

**Published:** 2026-09-23

**Authors:** Nazia Nazam, Sorina N. Shirley, Joel C. Opara, Shamza Manzoor, Maryam G. Shaikh, Morgan L. Brown, Pranava Nande, Ali M. Eakes, Abdulraheem Kaimari, Jianmei W. Leavenworth, Michael Ohlmeyer, Elizabeth A. Beierle

**Affiliations:** 1 Division of Pediatric Surgery, Department of Surgery, University of Alabama at Birmingham, Birmingham, AL 35233, USA; nazianazam@gmail.com (N.N.);; 2 Department of Neurosurgery, University of Alabama at Birmingham, Birmingham, AL 35233, USA; 3 O’Neal Cancer Center, University of Alabama at Birmingham, Birmingham, AL 35233, USA; 4 Atux Iskay, LLC, Plainsboro, NJ 08536, USA; michael.ohlmeyer@gmail.com

**Keywords:** therapy-induced senescence (TIS), PP2A, topotecan, neuroblastoma, senescence-associated secretory phenotype (SASP)

## Abstract

**Background/Objectives:** Therapy-induced senescence (TIS), while initially growth-suppressive, may promote tumor recurrence through the persistence of senescent cells and the senescence-associated secretory phenotype (SASP). Topotecan, a topoisomerase inhibitor and a key component in the treatment of neuroblastoma (NBL), is known to lead to TIS. Protein phosphatase 2A (PP2A) is a serine/threonine phosphatase that has been linked to cellular senescence. PP2A is downregulated in NBL, primarily through upregulation of its endogenous inhibitors. We have previously shown that a novel small molecule, ATUX-1215, activates PP2A in NBL cells. **Methods:** The current study investigates the PP2A activator, ATUX-1215, as a senotherapeutic strategy in NBL. **Results:** We demonstrate that combining ATUX-1215 with topotecan leads to a reduction in senescent cell burden. Combination therapy suppressed PAI-1 and CCL2, key components of the SASP secretome, and enhanced apoptosis. PP2A activation reduced viability, stemness, and clonogenic survival post-TIS. In vivo, the combination of ATUX-1215 treatment with topotecan significantly delayed tumor growth and attenuated the pro-tumorigenic serum SASP. **Conclusions:** Our findings position PP2A activation as a senotherapeutic approach, transforming the tumor-promoting TIS program into a therapeutic vulnerability, offering a promising avenue to mitigate therapy resistance and improve outcomes in NBL.

## Simple Summary

Neuroblastoma is the most common childhood tumor occurring outside the brain. Children who have the disease return after their treatment is completed have the worst outcomes. There are many reasons why the cancer may come back after it is treated. One of the reasons is that neuroblastoma cells go into a resting state after chemotherapy. These resting cells secrete substances into and around the tumors that prevent the body from fighting the cancer cells. We found a drug that will decrease the ability of cancer cells to go into this resting state. This drug also stops the secretion of those substances that support the cancer cells. These findings were seen in cell culture studies and in animal studies. These findings could have a huge impact on how neuroblastoma is treated in the future.

## 1. Introduction

Neuroblastoma (NBL) is the most common extracranial tumor of childhood. Children with relapsed or refractory disease have realized nominal improvements in survival over the last three decades [1,2]. Senescence, a cell fate of growth arrest, is often seen after chemotherapy [3,4,5]. The previously held belief that senescence is a permanent, non-proliferative barrier against tumorigenesis, is now challenged by evidence of its dynamic and reversible nature, carrying major implications for therapy-induced senescence (TIS)-based treatments in NBL [5]. Topoisomerase inhibitors, such as topotecan, are standard agents used for high-risk and recurrent NBL treatment, but these agents have been shown to lead to TIS. These dormant NBL cells may evade therapy, conferring poor therapeutic response and disease relapse [5]. In addition, the senescent cells display a senescence-associated secretory phenotype (SASP) in their secretome, involving factors that may either promote or suppress the tumorigenic phenotype [6].

Recent studies have demonstrated the targetability of senescent NBL tumor cells [5,7]. Suggested options include BCL-XL inhibitors to promote apoptosis and thereby reduce senescent cells. The compound A1331852 is an example of this strategy in NBL [8]. Herter et al. showed that BCL-2 inhibitors combined with MEK inhibition were effective in targeting senescent NBL cells [7]. Further, in other preclinical NBL models, venetoclax, a BCL-2 family inhibitor, has demonstrated synergistic activity with topotecan [9]. There are limitations to these approaches. Venetoclax is constrained by dose-limiting thrombocytopenia, and the efficacy of BCL-2–targeting senolytics varies across cell types and senescence triggers [10]. The use of senomorphic agents such as mTOR, JAK, or p38 MAPK inhibitors suppresses SASP production but does not clear senescent cells and may require prolonged administration [11]. These limitations highlight the need for alternative senotherapeutic strategies in high-risk NBL that simultaneously abrogate the SASP and drive senescent tumor cell death. Therefore, the ideal novel combinatorial strategies would first induce senescence and then follow with a senolytic agent to eliminate senescent cells, or a senomorphic agent to mitigate the pro-tumorigenic effects of the SASP [5,12].

Protein phosphatase 2A (PP2A) is a serine threonine phosphatase with known tumor-suppressor properties [13,14,15,16], but it is inactivated in many solid tumors, including NBL [17,18,19]. Emerging evidence links loss of PP2A activity to enhanced cellular senescence and disease progression. Studies have demonstrated that Alzheimer dementia, chronic obstructive pulmonary disease, and idiopathic pulmonary fibrosis are related to a decline in PP2A activity and an increase in cellular senescence [20,21,22,23,24]. Given the data supporting the role of PP2A in modulating senescence [25,26,27], its pharmacologic activation emerges as a promising senotherapeutic approach. However, the therapeutic potential of PP2A reactivation as a strategy to target TIS in NBL remains unexplored and provides the rationale for the focus of the current study.

## 2. Materials and Methods

### 2.1. Administered Reagents

Topotecan (CAS No. 123948-87-8, Selleckchem, Houston, TX, USA) was used to achieve TIS. Stock solutions (1 mM) were prepared in 100% DMSO and stored at −20 °C.

The PP2A activator, ATUX-1215, was prepared by methods detailed in Ohlmeyer patent application WO2023/023594. The chemical structure and the pharmacokinetic and pharmacodynamic data for ATUX-1215 have been reported [28,29]. ATUX-1215 has >98% purity per NMR, MS, and LC characterization. Concentrations of ATUX-1215 used for this study are based upon previously reported lethal dose 50% concentrations [30].

### 2.2. Cell Lines, PDXs, and Culture Conditions

SK-N-AS (MYCN-non-amplified; CRL-2137; female; RRID:CVCL_1700) and SK-N-BE(2) (MYCN-amplified, CRL-2271; male; RRID:CVCL_0528) human neuroblastoma cell lines were obtained from the American Type Culture Collection (ATCC, Manassas, VA, USA) and maintained under standard cell culture conditions previously described [30]. All cell lines were verified within the last 12 months with short tandem repeat (STR) analysis (Genomics Core, University of Alabama at Birmingham (UAB), Birmingham, AL, USA) and were free of Mycoplasma infection (Universal Mycoplasma Detection Kit, 30-1012K, ATCC).

### 2.3. Antibodies

Details of primary antibodies are in Table A1.

### 2.4. Extracting RNA, Preparing Library, and Sequencing

SK-N-BE(2) cells were treated with ATUX-1215 (10 µM) for 24 h, and cellular RNA was isolated (RNeasy kit, Qiagen Inc., Germantown, MD, USA) from two biologic replicates of each condition. Quality control, library preparation, and sequencing were completed by UAB genomics core. RNA integrity was ensured using the Agilent 2100 Bioanalyzer (Agilent Technologies Inc., Waldbronn, Germany), followed by two rounds of Poly A + selection and cDNA synthesis. The NEBNext Ultra Directional RNA Library Prep Kit for Illumina (New England Biolabs, Ipswich, MA, USA) was used for library preparation. Libraries were quantified with qPCR in a Roche LightCycler 480 with the Kapa Biosystems kit (Kapa Biosystems, Woburn, MA, USA). Sequencing was completed using the Illumina NextSeq500 platform incorporating the latest versions of the sequencing.

### 2.5. RNA Sequencing Analysis

RNA-seq reads were aligned to the human reference genome (GRCh38/hg38) using STAR (v2.7.10a; RRID:SCR_004463), and gene-level read counts were generated for downstream analysis. Lowly expressed genes with fewer than 10 total counts across all samples were removed prior to differential expression analysis. Differential expression was performed in R using DESeq2 (v1.44.0; RRID:SCR_000154), with control samples specified as the reference condition. Default DESeq2 normalization was applied, and *p*-values were adjusted for multiple testing using the Benjamini–Hochberg procedure. Differentially expressed genes were defined using thresholds of log2 fold change > 1 and adjusted *p*-value < 0.05. Analyses were restricted to protein-coding genes.

Targeted gene set enrichment analysis was performed using selected MYCN-related gene sets curated from MSigDB (v25.1.1) C2 (accessed 18 December 2025). Enrichment was evaluated using fgsea (v1.30.0) on a ranked gene list ordered by log2 fold change. All visualizations were generated in R with ggplot2 (v3.5.2; RRID:SCR_014601, accessed on 18 December 2025). Analyses were performed using publicly available packages. Raw and processed RNA-Seq data were deposited into the NCBI Gene Expression Omnibus (GEO) database under accession number GSE317934.

### 2.6. Viability

AlamarBlue assays (Thermo Fisher Scientific, Inc., Foster City, CA, USA) assessed viability. SK-N-AS and SK-N-BE(2) (1.5 × 10^4^) cells were plated in 96-well plates and treated with topotecan (0–14 μM for 24 h (SK-N-AS) and 72 h (SK-N-BE(2)). Then, alamarBlue dye (10 μL) was added, and the plates were read in a microplate reader (Epoch Microplate Spectrophotometer, BioTek Instruments, Winooski, VT, USA) for absorbance at 570 nm, keeping 600 nm as a reference wavelength. Experiments were completed in biological triplicate, and viability was reported as fold change ± SEM.

### 2.7. Synergy

SK-N-AS or SK-N-BE(2) cells (1.2 × 10^4^) were treated with either topotecan alone, ATUX-1215 alone, or a combination of both for 72 h. The half maximal inhibitory concentration (IC_50_) for the drug combinations and for each drug alone were calculated and isobolograms constructed using the Chou and Talalay method [31]. CI values of less than 1 indicated synergy, with those equal to 1 indicating an additive, and those more than 1 an antagonistic effect [31].

### 2.8. Immunoblotting

Whole-cell or fractionated cytoplasmic and nuclear lysates were utilized for immunoblotting as previously described [30,32]. Whole-cell lysates were prepared in RIPA buffer supplemented with protease inhibitor (Sigma Aldrich, Burlington, MA, USA), phosphatase inhibitor (Sigma Aldrich), and phenyl-methane sulfonyl fluoride (PMSF) (Sigma Aldrich). For fractionated lysates, cells were trypsinized; washed with PBS; and resuspended in cytoplasmic extraction buffer (10 mM HEPES, pH 7.9, 1.5 mM MgCl2, 10 mM KCl, 0.5 mM DTT, 1 mM EDTA, and 0.05% NP-40) supplemented with protease inhibitor (Sigma Aldrich), phosphatase inhibitor (Sigma Aldrich), and PMSF (Sigma Aldrich). Following incubation on ice and detergent-mediated lysis, the samples were centrifuged at 800× *g* for 4 min at 4 °C, and the supernatant, cytoplasmic fraction, was collected. The remaining pellet was resuspended in nuclear extraction buffer (5 mM HEPES, pH 7.9, 1.5 mM MgCl2, 300 mM NaCl, 0.5 mM DTT, 0.2 mM EDTA, and 26% glycerol) supplemented with protease inhibitor (Sigma Aldrich), phosphatase inhibitor (Sigma Aldrich), and PMSF (Sigma Aldrich) and vortexed every 5 min for 30 min to enable nuclear lysis, followed by centrifugation at 25,000× *g* and 4 °C for 15 min to obtain the nuclear fraction. Purity was confirmed by immunoblotting for GAPDH (cytoplasmic) and Histone H3 (nuclear). Precision Plus Protein Kaleidoscope Standards molecular-weight marker (1610375, Bio-Rad, Hercules, CA, USA) confirmed expected size of target proteins. Antibodies were used following the manufacturers’ protocol, and internal controls were included to ensure equal protein loading.

### 2.9. Senescence and DNA-Damage Markers

Senescence-associated β-galactosidase (SA-β-gal) staining marks senescent cells by detecting β-galactosidase activity [33]. SK-N-AS (4 × 10^4^) cells were plated in 12-well plates and treated for 72 h with topotecan (1.5 or 2.5 nM) and ATUX-1215 (5 µM) alone or in combination. SK-N-BE(2) (1 × 10^5^) cells were similarly plated and treated with 1 nM topotecan and 5 µM ATUX-1215 alone or in combination. At 72 h, growth media were removed, and fresh media were added. After 48 h, the cells were washed twice with PBS; stained using a SA-β-gal kit (#9860, Cell Signaling Technology, Danvers, MA, USA) per manufacturer’s protocol, with some modifications; and reported as signal intensity of SA-β-gal staining [34].

Phosphorylation at the C-terminal serine-139 of the H2AX histone [35,36], p21 expression [37], and total and phosphorylated CHK1 are recognized as markers of senescence. Detection of phosphorylated and total protein expression of H2AX, CHK1, and p21 was completed using immunoblotting.

### 2.10. Clonogenic Assay

Clonogenic assay was performed by seeding SK-N-AS and SK-N-BE(2) cells in a 12-well plate (1000 cells/well for SK-N-AS and 800 cells/well for SK-N-BE(2)), followed by treatment with either 5 μM ATUX-1215 or topotecan (2.5 or 5 nM in SK-N-AS and 0.3 or 0.5 nM in SK-N-BE(2)) alone or in combination. Plates were maintained in culture for 14–16 days and then fixed and stained with 0.5% crystal violet in 10% methanol. Colonies were quantified using ImageJ software (https://imagej.net/ij/, Ver 1.49, accessed on November 2024–April 2025).

### 2.11. ELISA

ELISA kits for human SERPINE1 (plasminogen activator inhibitor 1, PAI-1 (#HUFI00386, AssayGenie, Dublin, Ireland)) and human monocyte chemotactic protein 1 (MCP1)/C-C ligand 2 (CCL2) (#HUFI00196, AssayGenie) were used to investigate the SASP secretions in the cell supernatants and animal serum. For cells, topotecan or ATUX-1215 and their combination were tested in SK-N-AS or SK-N-BE(2) cells at similar concentrations described above. Briefly, for supernatant ELISA, 1 × 10^6^ cells were seeded into a 6-well plate with 2 mL of culture media for 6 h. Cells were treated for 72 h, media were replenished with an equal volume of fresh media, and cells were cultured for another 48 h. The resulting conditioned media were used to detect the SASP markers. For animals, serum was harvested at the time of euthanasia, and the same kits were used to detect SASP markers.

### 2.12. Quantitative Real-Time PCR (RT-qPCR)

The mRNA abundance of neuronal stemness markers was measured with RT-qPCR and included octamer-binding transcription factor 4 (OCT4), sex-determining region Y-box2 (SOX2), NANOG, and NESTIN. Cellular RNA was extracted (RNeasy kit, Qiagen), and cDNA was synthesized (iScript cDNA Synthesis kit, Bio-Rad). SsoAdvanced SYBR Green Supermix (Bio-Rad) was utilized for qPCR. Probes to *OCT4*, *SOX2*, and *NANOG* were from Invitrogen (10336022, ThermoFisher Scientific, Inc., Waltham, MA, USA), and for *NESTIN*, from Invitrogen (ThermoFisher Scientific) (forward—TCCAGGAACGGAAAATCAAG; reverse—GCCTCCTCATCCCCTACTTC). qPCR was performed with 10 ng cDNA in 20 μL reaction volume using an Applied Biosystems 7900HT cycler (Applied Biosystems, Thermo Fisher Scientific) with cycling conditions of 95 °C for 2 min, followed by 39-cycle amplification at 95 °C for 5 s and 60 °C for 30 s. Abundance of mRNA was analyzed in triplicate and normalized to *GAPDH* internal control. The ΔΔCt method was used to calculate gene expression [38], reported as mean fold change ± SEM.

### 2.13. Animal Statement

The University of Alabama at Birmingham (UAB) Institutional Animals Care and Use Committee (IACUC-09803) approved all animal experiments, and the studies were conducted within institutional, national, and NIH guidelines and in compliance with the Animal Research: Reporting of In Vivo Experiments (ARRIVE) guidelines. The UAB IACUC has a maximal tumor size of 2000 mm^3^, which was not exceeded in these studies.

### 2.14. In Vivo Tumor Growth

SK-N-BE(2) (1.8 × 10^6^, 25% Matrigel (BD Biosciences, San Jose, CA, USA)) cells were injected into the right flank of 6-week-old female athymic nude mice (RRID:MGI:5652489; Fredricks, Charles River, Wilmington, MA, USA). Tumors were measured with a caliper, and tumor volumes calculated ((width^2^ × length)/2, width the smaller measurement). When tumors reached 100 mm^3^, all animals were treated with topotecan (1 mg/kg, oral gavage; og) once daily for five days. Following topotecan treatment, animals were randomized using an online randomization tool (https://www.gigacalculator.com/randomizers/randomizer.php, accessed on 10 February 2025 and 25 February 2025) to receive 100 μL og twice daily of either vehicle (N,N286 dimethylacetamide (DMA, 271012, Sigma Aldrich, and Kolliphor HS 15, Solutol, 42996, Sigma Aldrich, n = 12; RRID:SCR_008988) or ATUX-1215 (75 mg/kg in DMA and Solutol, n = 11) [28]. Animals were weighed weekly. Animals were humanely euthanized 15 days following treatment initiation, or when IACUC parameters were met. Animal euthanasia was performed with CO_2_ in their home cages, followed by cervical dislocation as a secondary method. Tumor tissue and serum were collected at euthanasia. Prior to initiating the studies, a power analysis was completed using an online tool (https://www.gigacalculator.com/calculators/power-sample-size-calculator.php, accessed on 3 January 2025). Investigators were unblinded to the treatment groups.

### 2.15. R2-Database Analysis for NBL Dataset

R2 Genomics Analysis and Visualization Platform was accessed for mRNA expression data from 249 primary human NBL samples (Tumor Neuroblastoma TARGET Asgharzadeh-249-custom-huex10) to complete gene-expression comparisons [39].

### 2.16. Statistical Tools and Analyses

Experiments were performed with at least three biologic replicates. Data were reported as mean ± SEM, as deemed appropriate. The statistical analyses, including ANOVA, two-tailed *t*-test, and Kaplan–Meier curves with log rank statistics, were completed using GraphPad Prism (Version 10.0.2, accessed on June 2024–August 2026; RRID:SCR_002798).

## 3. Results

### 3.1. Topotecan Induced TIS in NBL

We first wanted to confirm that topotecan induces TIS in the NBL cells. Topotecan treatment reduced cell viability, but a substantial fraction of metabolically active, non-proliferating cells remained after treatment consistent with TIS (Figure S1A,B; left panels). Treated cells showed increased SA-β-gal staining and elevated γH2AX levels (Figure S1A,B; middle and right panels, respectively). These assays verify topotecan-induced TIS in both SK-N-AS and SK-N-BE(2) cells. Full-sized photomicrographs are available in Supplementary Figure S2A,B.

### 3.2. ATUX-1215 Reduces TIS

Since SK-N-BE(2) cells are MYCN-amplified, representing the most difficult NBL tumors to treat, we initially completed bulk RNA-sequencing with these cells, comparing untreated cells to those treated with ATUX-1215. RNA-seq data were analyzed with pathway analysis. Pathways most associated with the current study of interest are highlighted in Supplementary Figure S3 and included a significant downregulation in the reactome_cellular_senescence pathway (NES < –1.0) compared to untreated cells, providing the impetus to investigate the potential role for ATUX-1215 in inhibiting senescence in neuroblastoma.

Next, topotecan and ATUX-1215 were combined to assess the effects on viability and TIS. Isobolograms constructed from 72 h data showed combination indices (CIs) less than one, indicating synergy between ATUX-1215 and topotecan in SK-N-AS (Figure 1a) and SK-N-BE(2) (Figure 1d) cells. Adding ATUX-1215 decreased SA-β-gal intensity (TIS), reaching statistical significance in SK-N-AS (Figure 1b) and SK-N-BE(2) (Figure 1e) cells. Phosphorylated H2AX (γ-H2AX) is a marker of senescence [35,36]. Topotecan treatment increased γH2AX expression in SK-N-AS cells (Figure 1c, top panel), but when combined with ATUX-1215, this effect was abrogated (Figure 1c, top panel). Similar findings were noted in SK-N-BE(2) cells (Figure 1f, top panel).

Other senescence markers include increased p21 [37] and decreased pCHK1. Immunoblotting revealed increased p21 with topotecan treatment that decreased with the addition of ATUX-1215 in SK-N-AS cells (Figure 1c, middle panels) and also diminished in SK-N-BE(2) cells (Figure 1f, middle panels). In SK-N-AS cells, topotecan decreased pCHK1 that was reversed with the addition of ATUX-1215 (Figure 1c, bottom panels). Similar results were seen in SK-N-BE(2) cells (Figure 1f, bottom panels). Together, these data suggest that ATUX-1215 mitigates topotecan-induced senescence.

### 3.3. ATUX-1215 Reduces Proliferation and Stemness of Topotecan-Treated Cells

We next assessed clonogenic capacity. Topotecan or ATUX-1215 treatment alone did not significantly affect colony formation in the SK-N-AS cells (Figure 2a) or the SK-N-BE(2) cells (Figure 2c). Colony formation was significantly decreased in both cell lines with the combination treatment of topotecan and ATUX-1215 (Figure 2a,c).

Clonogenic assays reflect cancer-cell stemness. To further corroborate stemness, we evaluated the effects on stemness genes *OCT4*, *SOX2*, *NANOG,* and *NESTIN*. In SK-N-AS cells, mRNA abundance of *SOX2*, *NANOG*, and *NESTIN* were significantly upregulated with topotecan treatment (Figure 2b), and the addition of ATUX-1215 to topotecan-treated cells significantly reduced the mRNA abundance of *SOX2* and *NANOG* (Figure 2b). A similar trend was noted with *NESTIN*, but it did not reach statistical significance (Figure 2b). In SK-N-BE(2) cells, there was a general trend for upregulation of mRNA abundance of all four stemness genes with topotecan, but only OCT4 was statistically significant (Figure 2d), and it was significantly reduced with the addition of ATUX-1215 (Figure 2d).

### 3.4. ATUX-1215 Modulates SASP Markers

The protein expression of SASP markers MMP3, PAI-1, CCL2, IL-1β, and TNFα were evaluated in a pilot study to identify soluble factors that may drive the tumorigenic effects of TIS (Figure S4). PAI-1 and MCP1/CCL2 were increased with topotecan treatment (Figure S4). PAI-1 has been reported to play a role in NBL metastasis [40], and CCL2 has also been studied preliminarily in NBL [41,42]; hence, we chose these two SASP proteins for further study. Topotecan treatment in SK-N-AS cells led to significantly more PAI-1 (Figure 3a) and CCL2 (Figure 3b) in the media. The addition of ATUX-1215 decreased these secretory proteins, but it did not reach statistical significance (Figure 3a,b). In SK-N-BE(2) cells, the media from cells treated with topotecan had significantly increased levels of PAI-1 (Figure 3c) and CCL2 (Figure 3d) that were significantly reduced when ATUX-1215 was added (Figure 3c,d).

These findings steered us to investigate the relation of PAI-1 and CCL2 to NBL patient survival. We employed a publicly available dataset [39]. In *MYCN*-non-amplified (Figure 3e) patient samples, there was a statistically significant negative correlation between *PAI-1/SERPINE1* (gene encoding PAI-1) abundance and overall patient survival (Figure 3e, left panel). In this patient cohort, there was a trend toward decreased survival with increased *CCL2* abundance, but it did not reach statistical significance (Figure 3e, right panel). In *MYCN*-amplified patient samples, there was a statistically significant negative correlation between *PAI-1/SERPINE1* (gene encoding PAI-1) abundance (Figure 3f, left panel) and *CCL2/MCP1* (gene encoding CCL2) abundance (Figure 3f, right panel) and overall patient survival. Thus, ATUX-1215 decreased the increase in PAI-1 and CCL2 seen following treatment with topotecan, and lower levels of these SASP candidates are associated with improved overall survival in NBL patients.

### 3.5. ATUX-1215 Induces Apoptosis in Topotecan-Treated NBL Cells

We next investigated the cell fate toward apoptosis (caspase 3 cleavage) or survival (NF-κB/AKT phosphorylation). There was evidence of apoptosis with increased caspase 3 cleavage in both cell lines upon treatment with ATUX-1215 (Figure 4a,d). In SK-N-AS cells, the increased caspase 3 cleavage in the low combination (topotecan, 1.5 nM and ATUX-1215, 5 µM) corroborates with decreased activation of NF-κB (p65) promoting cell death (Figure 4a,b). Conversely, at a higher dose of topotecan (2.5 nM), the combination with ATUX-1215 resulted in increased NF-κB p65 (Figure 4b), supportive of survival and, hence, decreased caspase 3 cleavage (Figure 4a). Further, there was increased compensatory survival signaling through increased AKT phosphorylation in these cells treated with ATUX-1215 at a higher topotecan dose compared to lower topotecan dose (Figure 4c). These findings indicate that PP2A activation induces apoptosis-mediated cell elimination at lower doses of topotecan, but at higher topotecan doses, the increased pAKT and NF-κB p65 suggest a state where cells persist in a non-proliferative condition as evidenced by decreased senescence markers (Figure 1b,c) and SASP secretion (Figure 3a,b).

In SK-N-BE(2) cells, combination treatment resulted in increased apoptosis, as witnessed by caspase 3 cleavage in the combination versus topotecan alone (Figure 4d). With the combination, pNF-κB was decreased, indicative of apoptosis (Figure 4e). However, AKT phosphorylation was also inhibited. Either of the administered agents inhibited AKT signaling as single agent, while the combination decreased it even further (Figure 4f). Thus, in SK-N-BE(2) cells, PP2A activation appears to eliminate cells that may be in a topotecan-induced senescent state via apoptosis.

### 3.6. Topotecan Combined with ATUX-1215 Results in Reduced Tumor Volumes and Serum SASP Markers In Vivo

We next proceeded with in vivo studies. We employed SK-N-BE(2) cells since MYCN-amplified NBLs are the most difficult to treat. SK-N-BE(2) cells were injected into the flank of athymic nude mice. When tumors reached 100 mm^3^, all animals were treated (days 1–5) with topotecan. Animals were subsequently randomized into two groups: vehicle and experimental. The vehicle group received DMA (vehicle, n = 12), and the experimental group received ATUX-1215 (75 mg/kg (og) twice daily, n = 11) (Figure S5, lower panel), based on previously published pharmacokinetic data [30]. As shown in Figure 5a, ATUX-1215 treatment significantly decreased tumor volume starting day 8, compared to vehicle-treated animals. Animals bearing topotecan-treated SK-N-BE(2) tumors had a significant decrease in relative tumor growth (Figure 5b) and took a longer time for tumors to reach 2000 mm^3^ (Figure 5c) when comparing those treated with ATUX-1215 to those treated with vehicle.

Immunoblotting on tumor-tissue lysates showed decreased SA-β-gal expression in the ATUX-1215 group (Figure 5d). In general, treatment with ATUX-1215 decreased expression of PAI-1 and CCL2, γH2AX, and increased caspase 3 and cleaved PARP (Figure 5d). PAI-1 and CCL2 (Figure 5e,f, respectively) were significantly decreased in the serum of ATUX-1215-treated animals compared to vehicle-treated animals. These results suggest that ATUX-1215 may act on established senescent cells rather than preventing senescence, reducing tumor growth through increased apoptosis and reduced SASP.

## 4. Discussion

We have previously shown that the small molecule, ATUX-1215, activates PP2A in established NBL cell lines and patient-derived xenograft cells [30]. The current study shows that ATUX-1215 functions as a senotherapeutic in NBL, counteracting topotecan-induced senescence. Other investigators have noted similar effects with PP2A activation. Li and colleagues showed that combining topotecan with FTY720, an immunomodulatory drug known to activate PP2A, increased NBL tumor cell death and decreased tumor growth in animals bearing NBL tumors [43]. The current study advanced to investigate the mechanisms of these findings.

We noted in our bulk RNA-sequencing findings that ATUX-1215 treatment led to the suppression of cellular processes and gene-expression patterns associated with senescence, and although we did not have sequencing data on the effects of topotecan alone on the pattern of expression of senescence genes, we felt that the sequencing data comparing ATUX-1215-treated to -untreated cells provided adequate impetus to study PP2A activation in the setting of treatment-induced senescence.

ATUX-1215 treatment was synergistic with topotecan in both MYCN-amplified and -non-amplified NBL cells. However, functional assays added that MYCN-amplified cells demonstrated changes in their phenotype at lower concentrations of topotecan than non-amplified cells. This observation aligns with previous findings where MYCN-amplified NBL cells were found to be highly sensitive to topotecan, and non-amplified cells were seen maintaining normal proliferation at similar drug concentrations [44]. We also noted that the ability of ATUX-1215 to reduce senescence was greater in the amplified cells. This differential response may reflect MYCN-driven replication stress [45], enhancing vulnerability to PP2A activation.

Gamma-H2AX is a known marker of senescence [35,36], and evidence exists showing that PP2A activation may modulate senescence through γH2AX. Chowdhury et al. demonstrated that the catalytic subunit of PP2A directly binds with γH2AX, leading to dephosphorylation and resultant DNA-damage repair [46]. In our study, the addition of ATUX-1215 to topotecan-treated NBL cells reduced γH2AX. However, we also found sustained phosphorylated CHK1, which would indicate that the ATR-CHK1 axis is functioning and that loss of γH2AX does not reflect DNA-damage repair but represents a reversal of senescence. In addition, we found an increase in caspase 3 activation, and since caspase 3 is known to interrupt DNA-damage-repair responses, the changes in γH2AX may reflect elimination of selective cells via apoptosis or direct PP2A dephosphorylation of γH2AX. Nonetheless, interrupting H2AX in NBL is important.

Further evidence of the significance of interrupting H2AX in NBL is provided by Ognibene et al. They evaluated the gene-expression profiles of 786 NBL tumors and found that H2AFX overexpression was associated with poor patient survival irrespective of MYCN amplification [47]. We analyzed other publicly available datasets [39] and found that low H2AX correlates with improved overall survival in MYCN-non-amplified (Figure S6A), but not in MYCN-amplified patients (Figure S6B), which may reflect the small number of amplified patients in the dataset. These data reinforce PP2A activation as a therapy component in modulating TIS, irrespective of MYCN-amplification status.

We noted that NBL cells continued to form colonies after topotecan treatment. Since senescent cells are typically non-proliferative, this finding may reflect SASP-driven, pro-tumorigenic signaling. We observed an increase in SASP proteins following topotecan treatment, supporting that conclusion. In addition, we allowed cells a 48 h drug-free period post-treatment, so the emergence of a cell population released from senescence, known for higher tumor initiating capacity [48], may further explain the noted clonogenic growth. The observed reduction in colony formation seen with the addition of ATUX-1215 may reflect reduced TIS, reduced SASP signaling, and limited senescence escape.

PP2A functions partly through its suppressive effects on cancer cell stemness by dephosphorylating key stemness pathways, such as mammalian target of rapamycin (mTOR) [49,50,51] and NF-κB [52]. These findings are important in the context of stemness. Wang et al. demonstrated that AKT drives SOX2 [53], and Khalil and colleagues showed that AKT phosphorylation was related to stemness and Nanog, Oct-4, and Sox2 protein expression in NBL cell lines [54]. Additionally, NF-κB has been implicated in the transcriptional regulation of SOX2 and NANOG in various cancer contexts [53,55]. We found similar evidence in our studies that topotecan treatment induced markers of NBL stemness that were mitigated by the addition of ATUX-1215. We hypothesize, therefore, that PP2A activation, by attenuating these pathways, may suppress the transcriptional program driving topotecan-induced stemness. However, we acknowledge that our data may not fully establish this mechanism and alternatives, including selective elimination of stem-like cells or inhibition of de-differentiation, cannot be excluded. Future studies incorporating lineage tracing, chromatin accessibility analysis at promoters of stemness genes, and direct assessment of NF-κB/AKT transcriptional activity will be needed to resolve the precise mechanism.

Our study reveals a potential genetic-context dependency in the senotherapeutic action of PP2A activation. In MYCN-non-amplified cells, the effect of PP2A activation on cells treated with topotecan was dose-dependent. At low doses, NF-κB and AKT activation was suppressed, and caspase-3 cleavage enhanced. At higher doses, the cells remained arrested (SA-β-gal positive), with no significant increase in secreted PAI-1/CCL2, but with elevated pro-survival signaling (pAKT and pNF-κB) and less cleavage of caspase 3. This state of arrested cells without productive SASP or death, does not fit the senolytic or senomorphic paradigm [56]. We propose two non-mutually exclusive mechanisms: (i) phosphorylation–transcription decoupling of NF-κB, where p65 post-translational modifications or chromatin context prevent SASP-promoter access despite elevated phosphorylation [57], or (ii) a failed senolytic response, where insufficient PP2A-mediated suppression of AKT/NF-κB survival signaling leads to persistence rather than clearance of cells. In contrast, MYCN-amplified cells exhibited a clearer senolytic response, wherein the combination suppressed pro-survival AKT and NF-κB signaling and induced apoptosis via caspase-3/PARP cleavage, likely eliminating senescent cells. These findings align with known senotherapeutic mechanisms [58,59] and indicate the need for distinct clinical development strategies, prioritizing dose optimization for non-amplified tumors and validating the potent senolytic synergy in high-risk *MYCN*-amplified NBL. The functionality of cell-cycle checkpoint dysfunction may also contribute to these findings of dependency on genetic context. *MYCN*-amplified cells fail to arrest at the G1/S checkpoint [60]. Data suggest that the SASP only stops proliferation in cells with intact cell-cycle checkpoints. Therefore, the non-amplified SK-N-AS cells, with their intact checkpoints, remain in a non-proliferative state, while the SK-N-BE(2) cells are eradicated with apoptosis. The definitive explanation for these findings will require further investigations to discriminate between selective elimination of senescent cells and suppression of SASP without actual clearance of senescent populations. In this regard, long-term proliferation recovery assays and quantification of residual senescent cells after treatment may provide that clarification. Further, dissection of the mechanistic basis for these findings will require future investigation, such as ChIP for p65 at SASP promoters of PAI-1/CCL2 and determining p65 acetylation status.

Our in vivo data confirmed the in vitro results that demonstrated PP2A activation in the setting of topotecan treatment alters the SASP secretome in the *MYCN*-amplified SK-N-BE(2) cell line. SASP modulation may mitigate the pro-tumorigenic effects of senescence [5]. Given the link between high PAI-1/CCL2 and poor survival in high-risk *MYCN*-amplified patients, our findings position PP2A activators as a senotherapeutic strategy through the effects on SASP. In the *MYCN*-non-amplified cell line, SK-N-AS, the effects of PP2A activation on the SASP secretome in vitro were not as robust; it was decreased but failed to reach statistical significance. These findings warrant further evaluation of additional SASP markers [7,61,62] in future studies. Collectively, our data suggest that PP2A activation may mitigate key features of TIS in preclinical NBL models, raising the possibility that this strategy could complement existing chemotherapeutic regimens. However, definitive conclusions regarding clinical efficacy await validation in more advanced models and rigorous safety studies.

The significant reduction of serum CCL2 in vitro and in vivo following ATUX-1215 treatment carries immunological implications beyond direct tumor-cell targeting. CCL2 is a principal chemoattractant for monocytes, macrophages, and myeloid-derived suppressor cells (MDSCs), and its elevation in senescence drives pro-tumorigenic myeloid infiltration and immune evasion [6,63,64,65]. Since the athymic nude murine model retains functional macrophages, NK cells, and granulocytes despite lacking T cells [66], the suppression of systemic CCL2 following PP2A activation may disrupt blood-to-tumor myeloid recruitment, attenuating the inflammatory niche that sustains the persistence of senescent cells. This crosstalk of senescent tumor cells secreting CCL2, recruiting myeloid cells, and further promoting tumor growth represents an indirect but critical mechanism of SASP-mediated tumor progression. Whether ATUX-1215 directly reduces CCL2 from senescent tumor cells or indirectly alters the production by host immune cells remains unresolved and a limitation of the current study. We propose future studies incorporating longitudinal serum sampling to map SASP kinetics, and macrophage-depletion studies to test causality of the CCL2-myeloid axis in the anti-tumor efficacy of ATUX-1215.

We acknowledge that there are other limitations to these studies. The use of ATUX-1215 for PP2A reactivation may be associated with potential off-target effects. Since phosphatase activation often results in kinase inhibition, there may be an unwanted inhibitory effect on potential anti-tumor signaling pathways. These issues will be more deeply investigated in future studies using selective knock-out of the PP2A functional domain.

## 5. Conclusions

In the present preclinical study, we demonstrate that combining ATUX-1215 with topotecan suppresses the pro-tumorigenic SASP, decreases senescence-associated cells, and delays tumor growth (Figure S5), suggesting that PP2A activation may be a senotherapeutic strategy for NBL, potentially providing a mechanism to prevent tumor relapse and improve NBL outcomes. However, we acknowledge the limitations of our current mechanistic validation, and further studies including patient-derived xenograft models, combination therapies with standard-of-care regimens, and toxicity assessments will be required prior to clinical translation.

## Figures and Tables

**Figure 1:**
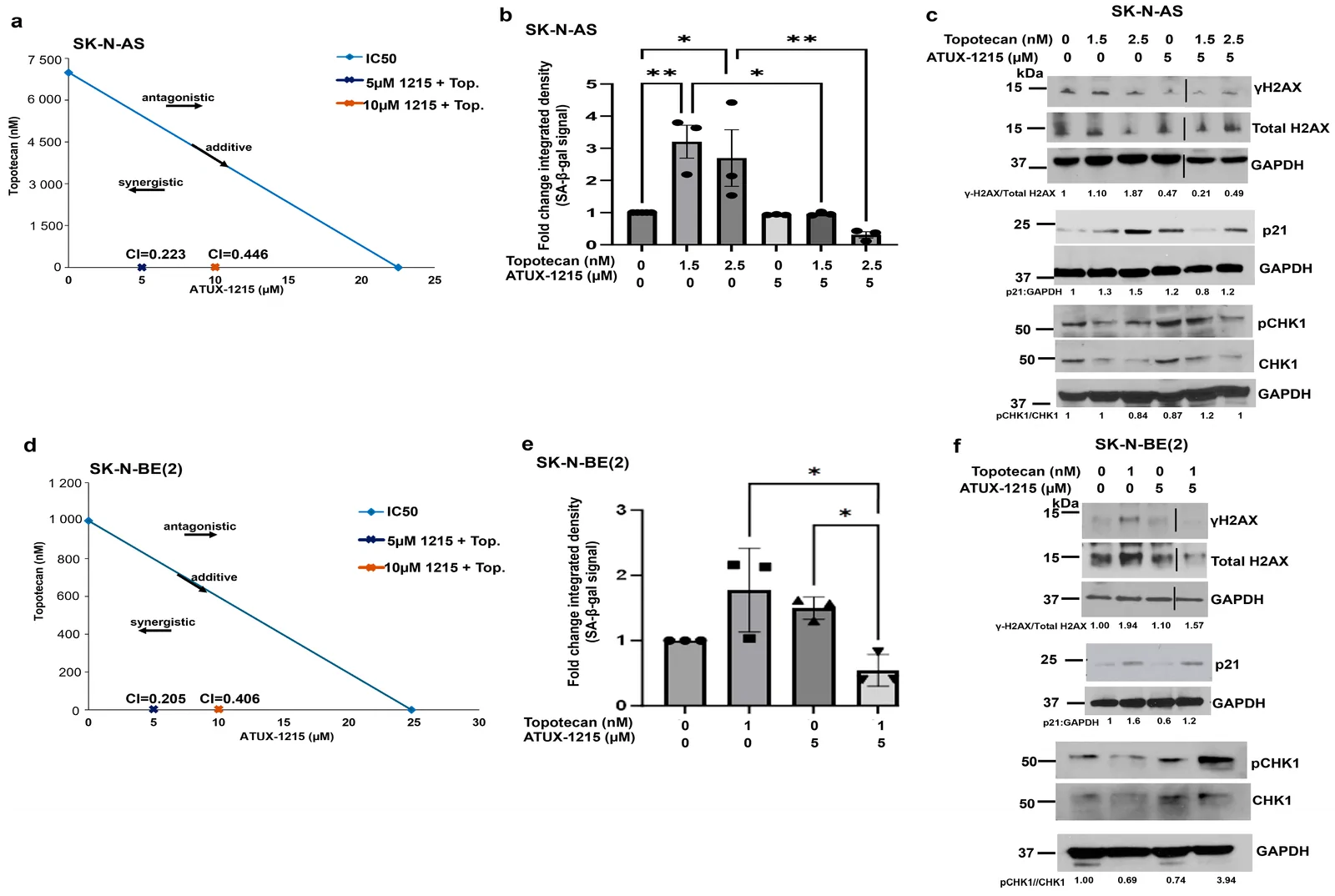
ATUX-1215 reduces TIS. (**a**,**d**) Isobolograms and combination indices (CI) in SK-N-AS (**a**) or SK-N-BE(2) cells (**d**). Cells (1.2 × 10^4^) were treated with either nothing, topotecan alone, ATUX-1215 alone, or their combination for 72 h. AlamarBlue assay detected viability and IC_50_ values calculated. CIs were calculated using the Chou–Talalay method [31]. Calculated CI values of less than 1 indicate synergy. (**b**,**e**) SA-β-gal staining was performed in SK-N-AS (4 × 10^4^) or SK-N-BE(2) (1 × 10^5^) cells treated with topotecan or ATUX-1215 alone or in combination for 72 h, followed by cell growth in fresh media for 48 h. SA-β-gal positive signal was significantly increased with topotecan in SK-N-AS (**b**) and SK-N-BE(2) cells (**e**). Addition of ATUX-1215 to topotecan significantly decreased SA-β-gal signal in SK-N-AS (**b**) and SK-N-BE(2) (**e**) cells. (**c**,**f**) Immunoblotting of whole-cell lysates demonstrates an increase in γH2AX following topotecan treatment that is diminished with the addition of ATUX-1215 (top panels). Similar findings were seen with p21 (middle panels). Topotecan treatment decreased pCHK1 in both cell lines that was abrogated with the addition of ATUX-1215 (bottom panels). GAPDH served as an internal loading control. Data represent at least three independent biologic replicates and are expressed as mean ± SEM. Statistical comparison was completed with one-way ANOVA. * *p* < 0.05; ** *p* < 0.01. SA-β-gal—senescence-associated β-galactosidase; Top—topotecan; 1215—ATUX-1215. The uncropped blots are provided at the end of the Supplementary Materials.

**Figure 2:**
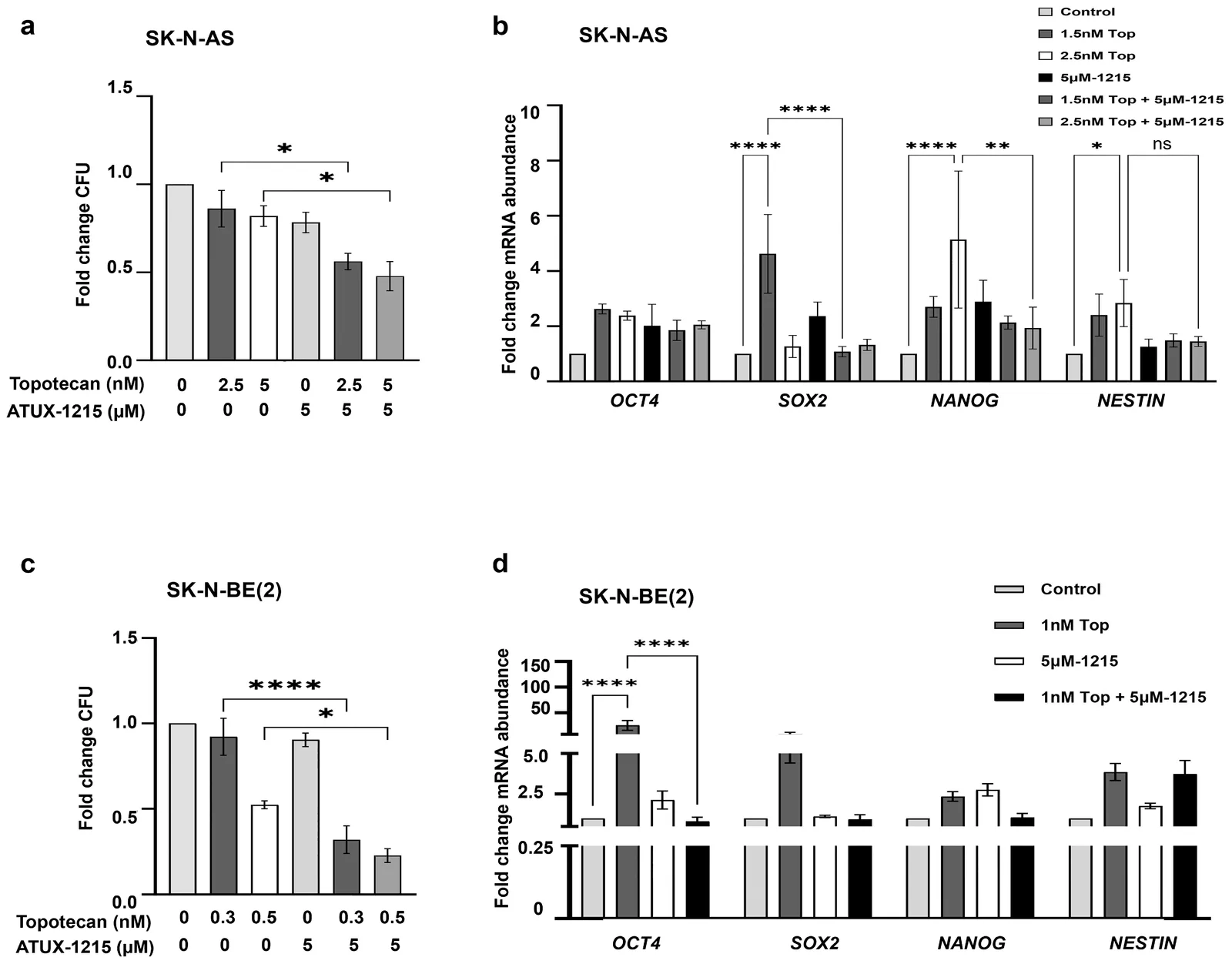
ATUX-1215 reduces proliferation and stemness in topotecan-treated cells. (**a**,**c**) Clonogenic assays were performed using SK-N-AS (**a**) and SK-N-BE(2) cells (**c**). (**a**) Topotecan or ATUX-1215 alone did not significantly reduce colony formation compared with untreated SK-N-AS cells. Co-treatment with ATUX-1215 significantly decreased colony formation compared to topotecan alone. (**c**) In SK-N-BE(2) cells, colony formation was significantly decreased with combination treatment compared to topotecan alone. (**b**,**d**) Quantitative RT-PCR analysis for OCT4, SOX2, NANOG, and NESTIN following the indicated treatments. (**b**) In SK-N-AS cells, topotecan significantly increased SOX2, NANOG, and NESTIN mRNA abundance, which was attenuated by ATUX-1215. (**d**) In SK-N-BE(2) cells, topotecan significantly upregulated OCT4, which was significantly diminished by ATUX-1215. Statistical comparison was completed with one-way ANOVA followed by Tukey’s multiple comparison test. ns—not significant; * *p* < 0.05, ** *p* < 0.01, **** *p* < 0.0001. CFU—colony-forming units; Top—topotecan; 1215—ATUX-1215.

**Figure 3:**
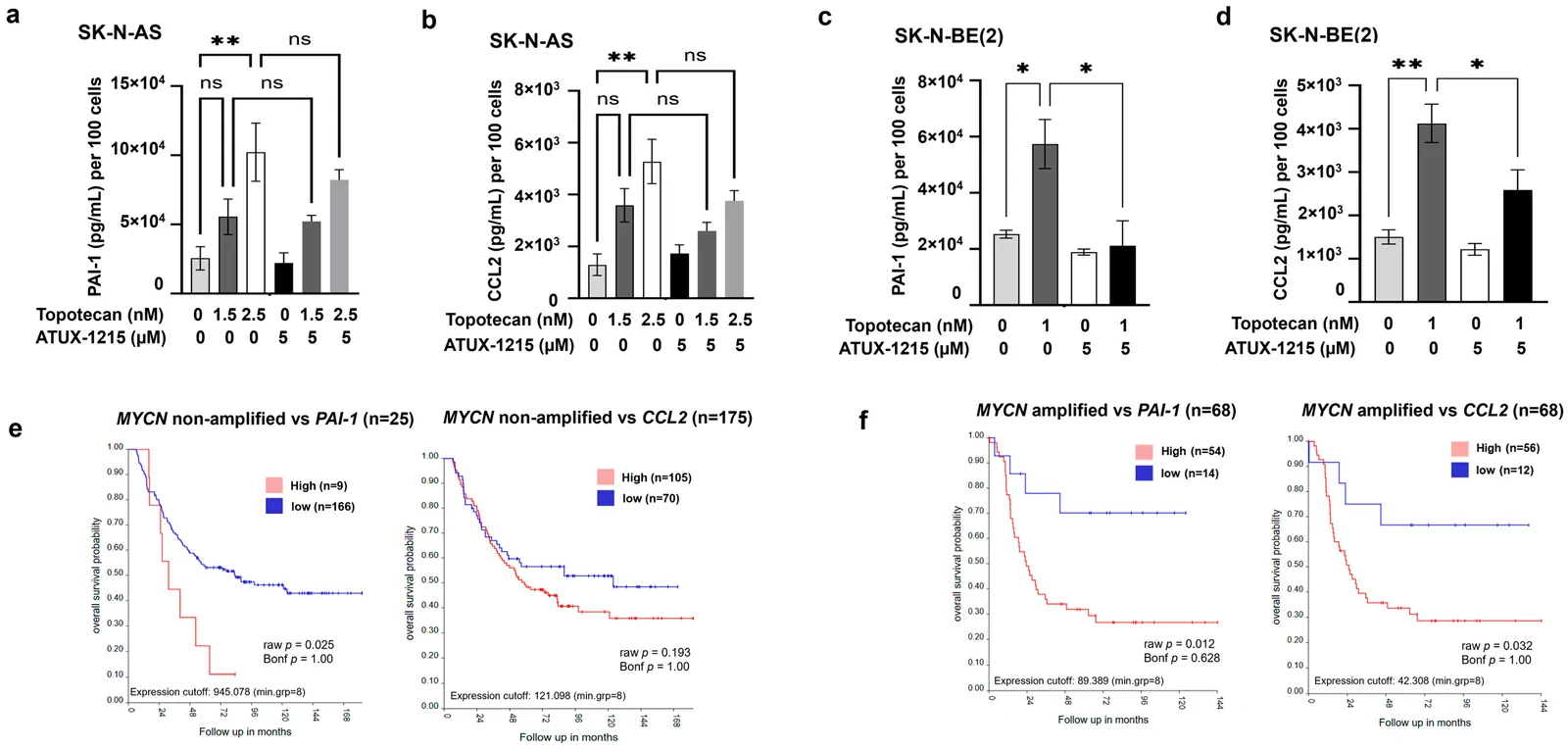
ATUX-1215 modulates SASP markers. (**a**–**d**) ELISA quantification of SASP factors, PAI-1 and CCL2, normalized to cell number (pg/mL per 100 cells). In SK-N-AS cells, topotecan increased secretion of PAI-1 (**a**) and CCL2 (**b**), which were reduced by ATUX-1215, although not reaching statistical significance. In SK-N-BE(2) cells, topotecan treatment significantly elevated PAI-1 (**c**) and CCL2 (**d**), and ATUX-1215 co-treatment significantly decreased their levels. (**e**,**f**) Kaplan–Meier survival curves and correlation analysis from the TARGET-Asgharzadeh dataset (n = 249). (**e**) In *MYCN* non-amplified samples, high mRNA expression of *PAI-1* (*SERPINE1*), but not *CCL2* (*MCP-1*), is associated with worse patient overall survival. (**f**) In *MYCN*-amplified patient samples, high *PAI-1* (*SERPINE1*) and high *CCL2* (*MCP-1*) are significantly associated with worse overall patient survival. Data represent mean ± SEM from three independent biological replicates. Statistical analysis was performed using one-way ANOVA, followed by Tukey’s multiple comparison test. ns—not significant, Bonf-Bonferroni correction; * *p* < 0.05, ** *p* < 0.01.

**Figure 4:**
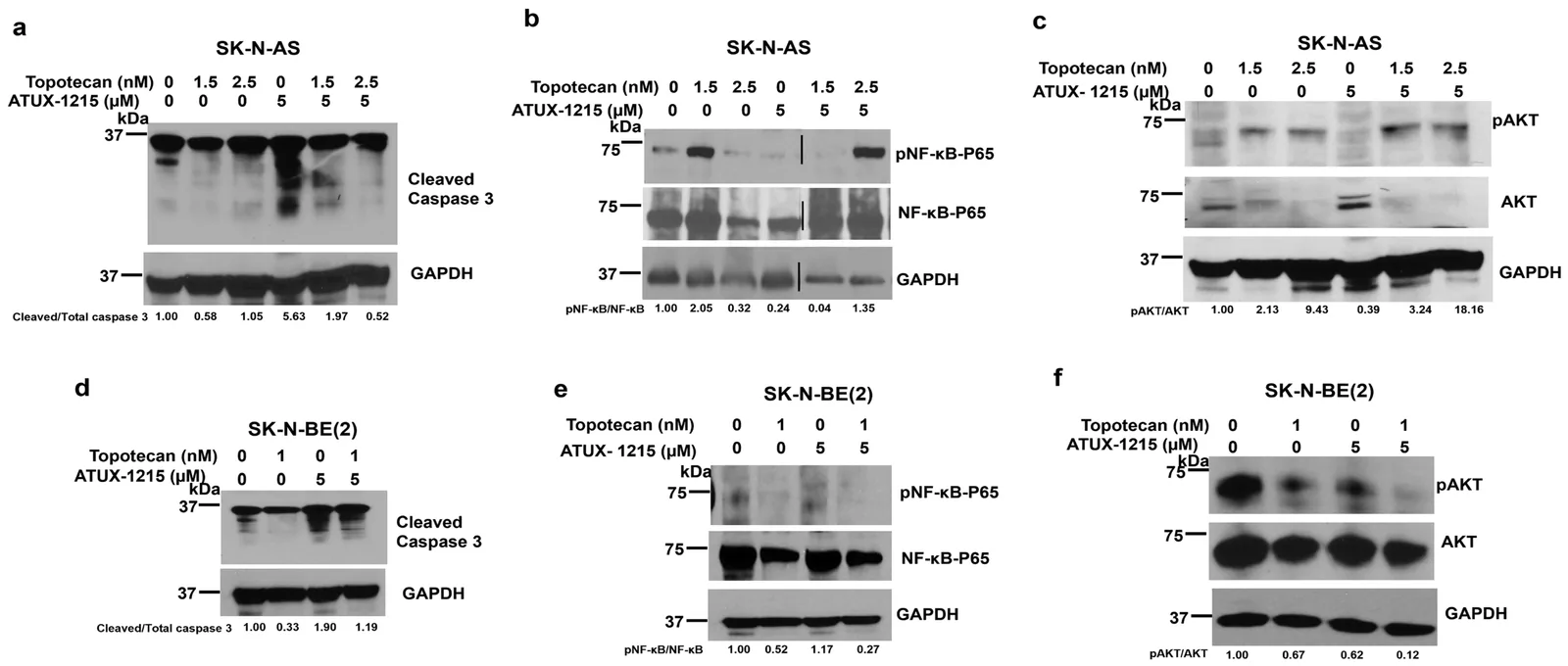
ATUX-1215 induces apoptosis in topotecan-treated NBL cells. (**a**–**c**) Immunoblotting was completed on lysates. (**a**) In SK-N-AS cells treated with topotecan and/or ATUX-1215, the low-dose combination (1.5 nM topotecan + 5 µM ATUX-1215) increased cleaved caspase-3 with reduced pNF-κB (p65) and enhanced pAKT (**a**–**c**). Higher topotecan (2.5 nM) combined with ATUX-1215 decreased cleavage of caspase 3 and increased pNF-κB and pAKT (**a**–**c**). (**d**–**f**) Immunoblotting was completed on lysates from SK-N-BE(2) cells treated with topotecan and/or ATUX-1215. Combination treatment increased caspase-3 cleavage (**d**) and reduced pNF-κB (**e**) and pAKT (**f**). GAPDH served as an internal loading control. The uncropped blots are provided at the end of the Supplementary Materials.

**Figure 5:**
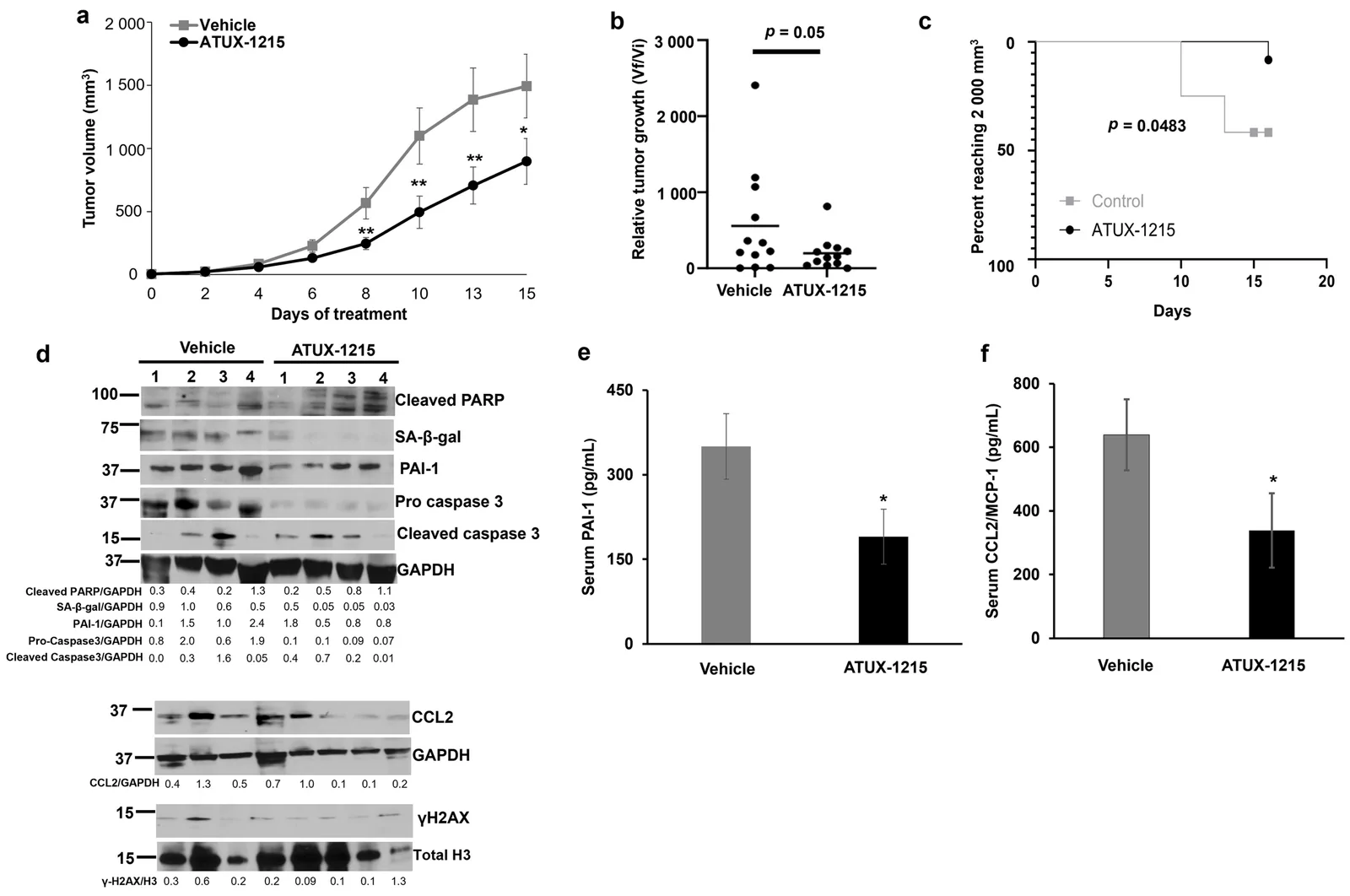
Topotecan combined with ATUX-1215 reduces tumor volume and serum SASP markers in vivo. Mice bearing SK-N-BE(2) tumors were treated with topotecan (days 1–5). Animals were randomized to receive vehicle (n = 12) or ATUX-1215 (n = 11). (**a**) Treatment with ATUX-1215 significantly reduced tumor volume from Day 8 onward compared to vehicle. (**b**) Endpoint relative tumor growth and (**c**) Kaplan–Meier analysis showed delayed tumor progression with the addition of ATUX-1215 treatment. (**d**) Immunoblotting of tumor lysates revealed decreased SA-β-gal, PAI-1, and CCL2; increased cleavage of caspase-3 and PARP; and a decrease in γH2AX in tumor lysates from ATUX-1215-treated animals. (**e**–**f**) Serum PAI-1 and CCL2 levels were significantly lower in serum from ATUX-1215-treated mice. Data represent mean ± SEM. Statistical analysis was performed using two-tailed Student’s *t*-test and Kaplan–Meier curves with log-rank statistics. * *p* < 0.05, ** *p* < 0.01. The uncropped blots are provided at the end of the Supplementary Materials.

## Data Availability

All data generated or analyzed in this study (including supplementary information files) are included in this article. These data are available from the corresponding author upon request. The RNA-Seq data were deposited into the NCBI Gene Expression Omnibus (GEO) database under accession number GSE317934.

## Supplementary Materials

The following supporting information can be downloaded at https://www.mdpi.com/article/10.3390/cancers18193088/s1, Figure S1: Topotecan induced TIS in NBL; Figure S2: Full-sized photomicrographs of SA-β-gal staining; Figure S3: ATUX-1215 reduces TIS; Figure S4: Screening of SASP factors; Figure S5: Schematic of the senotherapeutic action of PP2A activator, ATUX-1215; Figure S6: Association between *H2AFX* (*H2AX*) and overall survival in NBL patients.

## Abbreviations

The following abbreviations are used in this manuscript:

CCL2	Monocyte chemoattractant protein 1 (MCP1)
CFU	Colony-forming units
ChIP	Chromatin immunoprecipitation
CI	Combination indices
CHK1	Checkpoint kinase 1
MDSCs	Myeloid-derived suppressor cells
NBL	Neuroblastoma
PAI-1	Plasminogen activator inhibitor-1
PP2A	Protein phosphatase 2A
SASP	Senescence-associated secretory phenotype
SA-β-gal	Senescence-associated β-galactosidase
SEM	Standard error of the mean

## Appendix A

**Table A1:** Antibodies used in the current study.

Primary Antibodies	Company
γH2AX (2577S), Total H3 (4499S), CCL2 (39091S), PAI-1 (11907S), NF-κB P65 (8242S), phospho-NF-κB P65 (3033S), Cleaved caspase 3/Pro-caspase 3 (9496S), Cleaved PARP (5625S), AKT (9272S), phospho-AKT (4060S), total CHK1 (2G1D5, 2360S), phospho-CHK1 (S345, 2348S), p12(2946S); MMP2, IL-1β and TNF-α (SASP antibody sampler kit (38461))	Cell Signaling Technology (Danvers, MA, USA)
SA-β-gal (15518-1-AP)	Proteintech (Rosemont, IL, USA)
GAPDH (MAB374)	Millipore (Millipore Sigma, Rockville, MD, USA)
