# Supplementary material for: PP2A Activation as a Senotherapeutic in Neuroblastoma
Source: Cancers (Basel). 2026 Sep 23;18(19):3088. doi: 10.3390/cancers18193088 (PMC13664173; doi:10.3390/cancers18193088)

SUPPLEMENTARY FIGURES

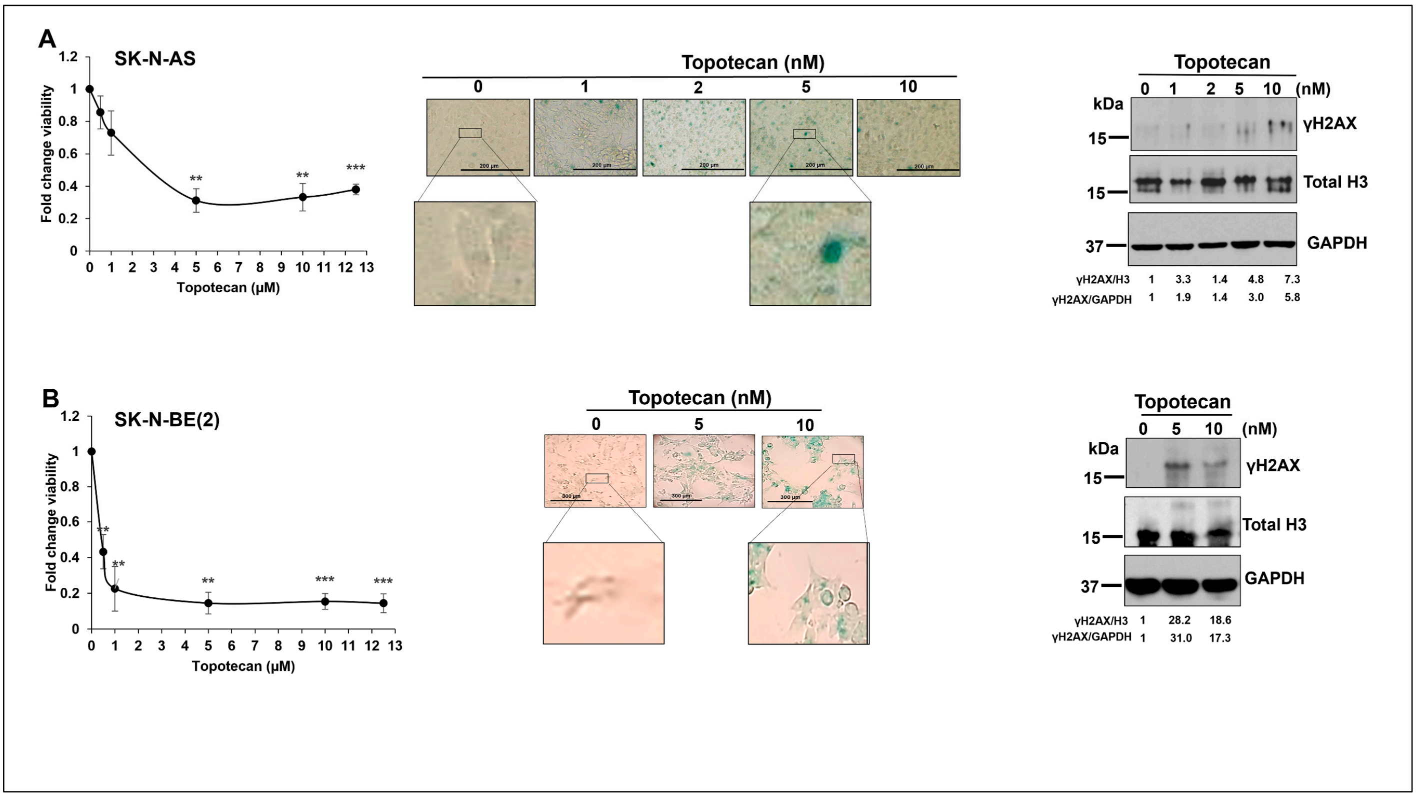

Figure S1. Topotecan induced TIS in NBL. A-B Cell viability was measured using alamarBlue assay in SK-N-AS and SK-N-BE(2) NBL cells. Cells (1.5×10^4) were plated in 96 well plates and treated with topotecan (0 - 12.5 μM) for 24 (SK-N-AS) or 72 (SK-N-BE(2)) hours. AlamarBlue dye (10 μL) was added, and the plates were read in a microplate reader at 570 nm, keeping 600 nm as a reference wavelength. Topotecan significantly affected viability but did not result in complete cell killing. Representative images of SA-β-gal staining of SK-N-AS (4×10^4) or SK-N-BE(2) (1×10^5) cells treated with topotecan (0 - 15 nM in SK-N-AS; 0 - 10 nM in SK-N-BE(2)) for 24 hours, showing control cells with minimal to no blue staining, indicating low senescence (**A**-**B**; *center panels*) with an increased SA-β-gal positive cells (blue cytoplasmic staining) observed with increasing topotecan doses (**A**-**B**; *center panel*). Immunoblots showing γH2AX expression (normalized to histone H3 or GAPDH), with enhanced expression in SK-N-AS (**A**; *right panel*) and SK-N-BE(2) (**B**; *right panel*) cells after treatment with topotecan. Data represent at least three independent biologic replicates and are expressed as mean ±

SEM. Scale bars represent 200 μm in **A** and 300 μm in **B**. Statistical comparison was completed with two-tailed Student's *t*-test. $^{**}p < 0.01$; $^{***}p < 0.001$. GAPDH or H3 served as internal protein loading controls.

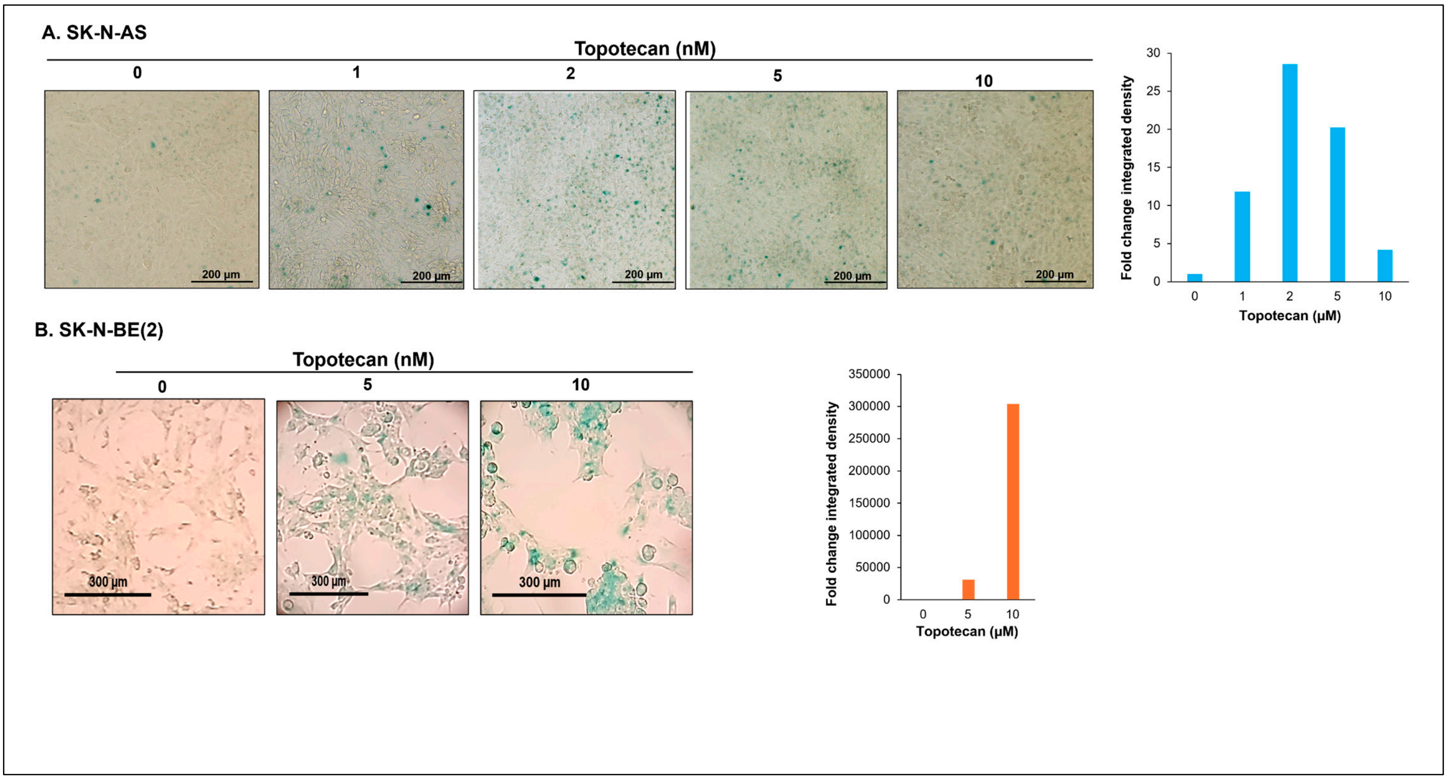

Figure S2. Full-sized photomicrographs of SA-β-gal staining. A-B Representative images of SK-N-AS and SK-N-BE(2) cells treated with topotecan to confirm TIS. SA-β-gal staining shows increased staining in topotecan treated SK-N-AS (0-15 nM) (**A**) and SK-N-BE(2) (0-10 nM) (**B**) cells compared to control. **A-B** SA-β-gal positive signal was quantified for each group of photomicrographs and is presented in graphic form beside the pictures. Scale bars: 200 µm for figure S2A and 300 µm for figure S2B.

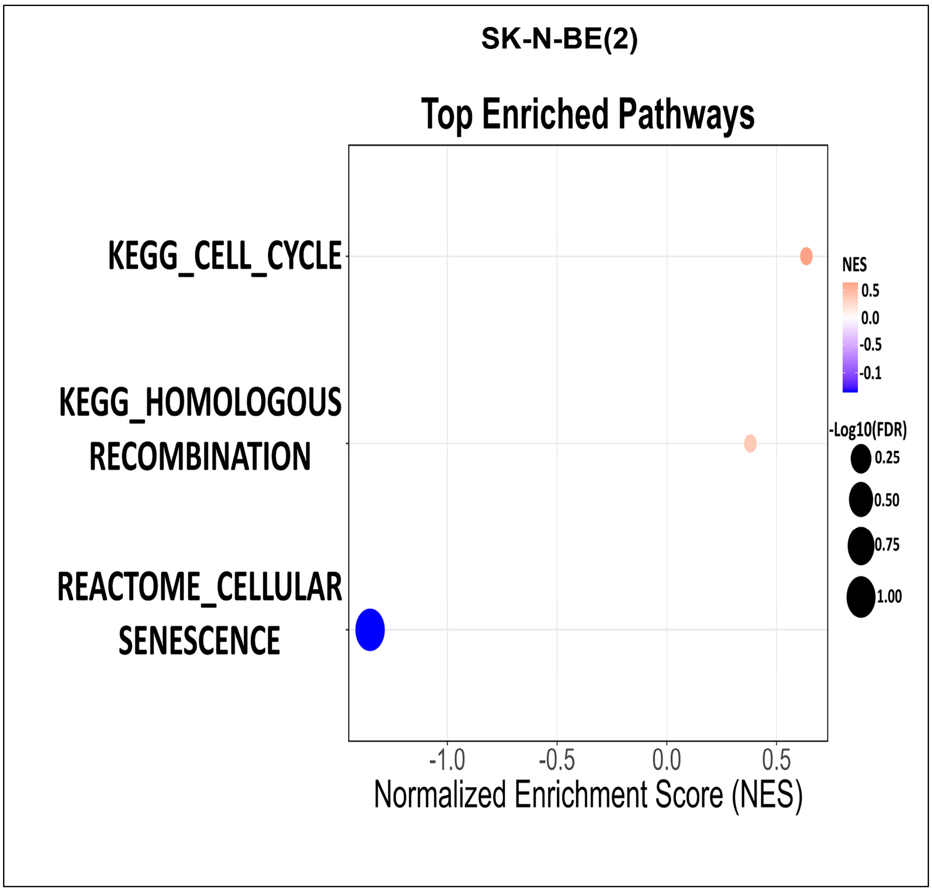

Figure S3. ATUX-1215 reduces TIS. Bulk RNA-sequencing from ATUX-1215 treated SK-N-BE(2) cells versus untreated cells indicate the reactome_cellular_senescence pathway to be significantly downregulated (NES < –1.0). GEO accession number GSE317934.

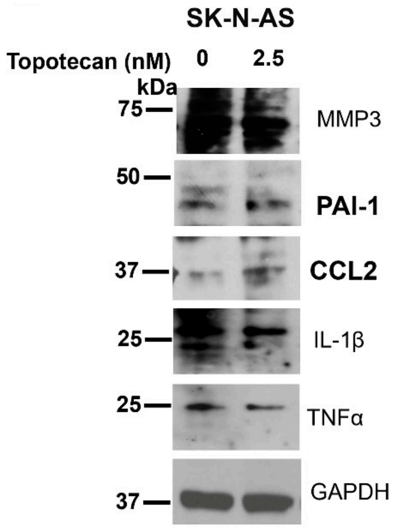

Figure S4. Screening of SASP factors. Immunoblot analysis of selected SASP proteins; MMP3, PAI-1, CCL2 (MCP-1), IL-1β, and TNFα, in SK-N-AS cells following treatment with topotecan (2.5 nM). Topotecan treatment affected the expression of several SASP components. GAPDH served as a loading control.

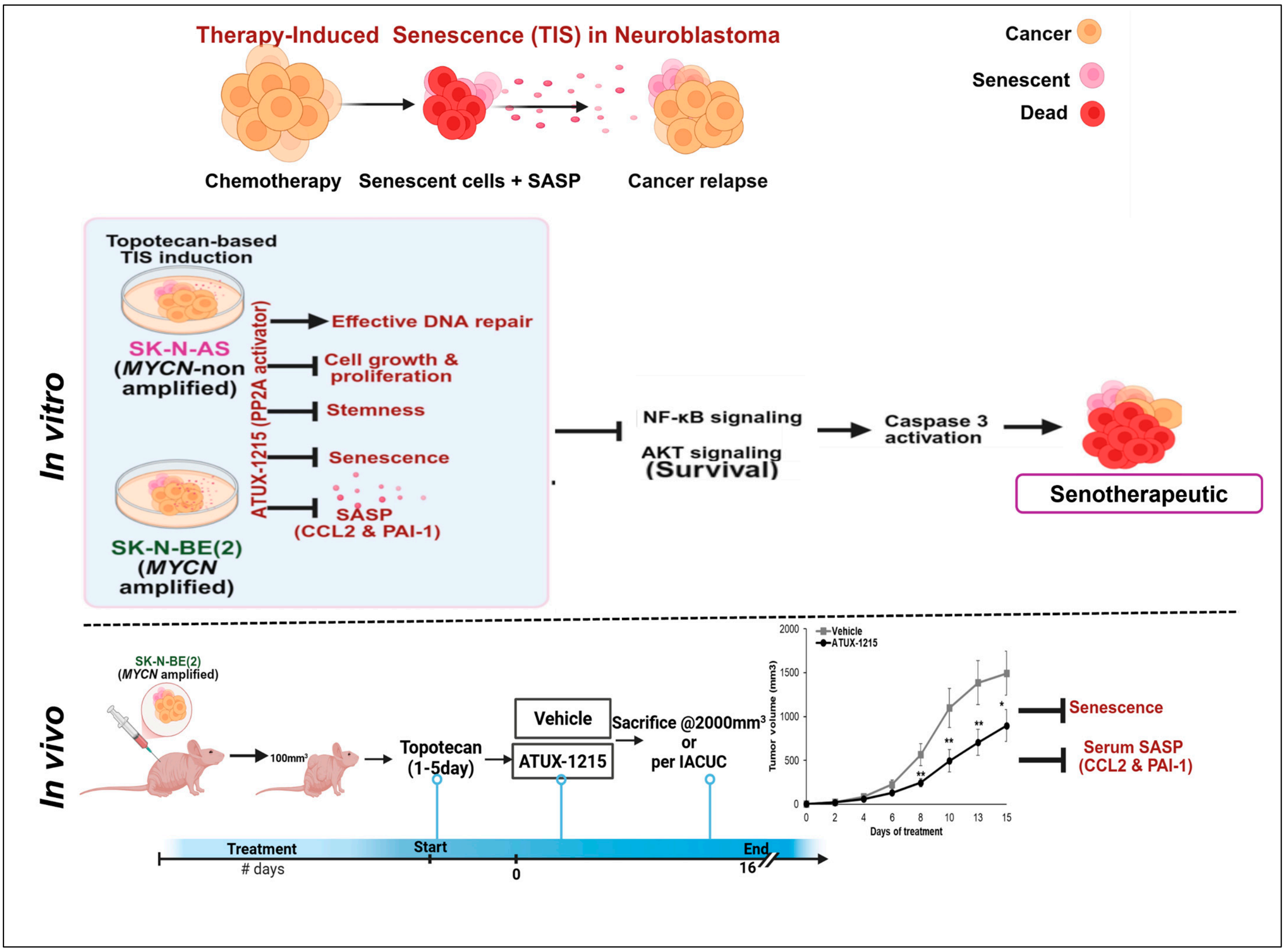

Figure S5. Schematic of the senotherapeutic action of PP2A activator, ATUX-1215. Topotecan monotherapy induces a senescent state characterized by SASP secretion which may fuel tumor relapse (*top panel*). The PP2A activator, ATUX-1215, works synergistically *in vitro* to transform this response, promoting effective DNA damage repair, inhibiting clonogenicity and cell growth *via* modulated AKT/NF-κB survival signals, suppressing SASP proteins, and triggering caspase-3-mediated apoptosis to eliminate senescent cells, thereby exerting a potent senotherapeutic effect (*middle panel*). Animals bearing SK-N-BE(2) tumors were treated with topotecan and then treated with vehicle or ATUX-1215. ATUX-1215 significantly reduced tumor volume, slowed tumor progression, and extended time to endpoint. Tumor lysates showed decreased SA-β-gal, PAI-1, CCL2, and γH2AX, with increased caspase-3 and PARP cleavage. Serum PAI-1 and CCL2 were also reduced, indicating lower senescence and SASP burden, supporting its senotherapeutic activity in *MYCN*-amplified NBL (*bottom panel*).

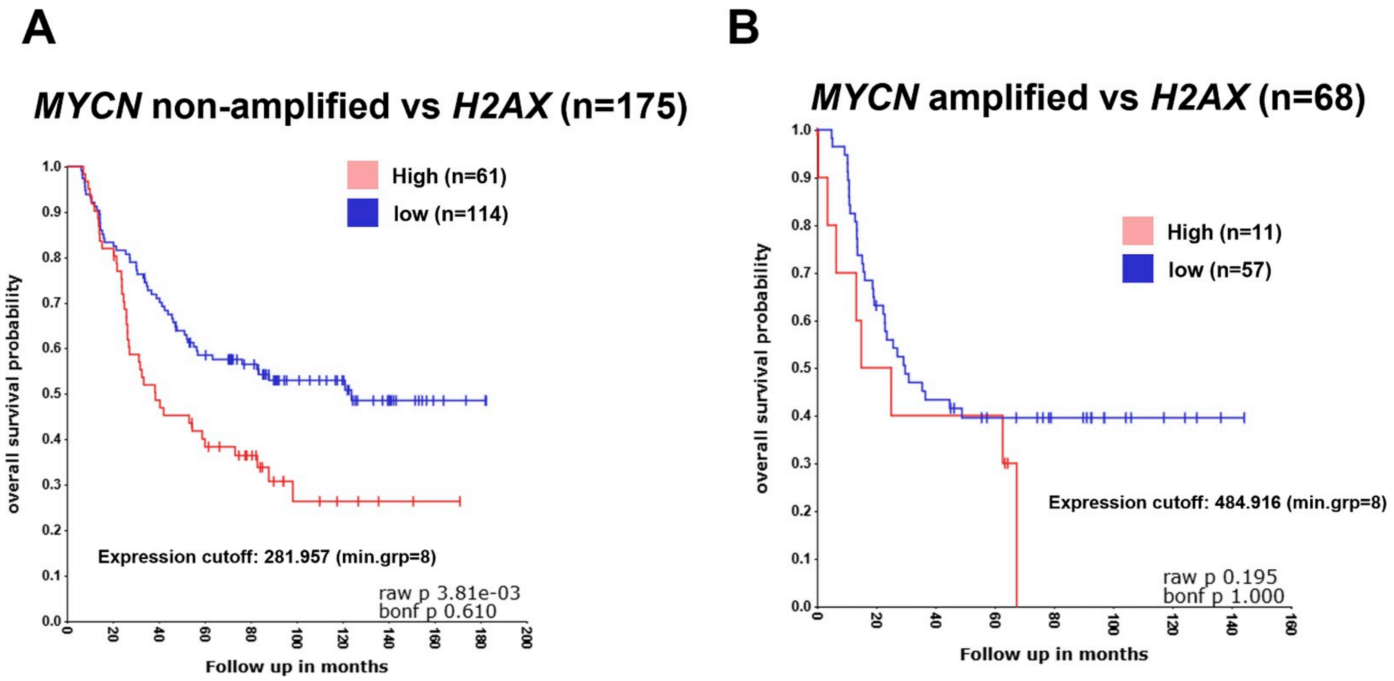

Figure S6. Association between *H2AFX* (*H2AX*) and overall survival in NBL patients. A-B Analysis of a publicly available NBL dataset (25) reveals low mRNA expression of the DNA damage marker *H2AFX(H2AX)* correlates with significantly improved patient survival in the *MYCN* non-amplified cohort (**A**) but did not reach statistical significance in the *MYCN* amplified cohort (**B**). This genetic context-dependency underscores the role of an efficient DNA damage response in favorable outcomes and supports the therapeutic strategy of enhancing this process via PP2A activation.

SK-N-AS

1C (top panels)

Topotecan (nM)	0	1.5	2.5	0	1.5	2.5
ATUX-1215 (μM)	0	0	0	5	5	5

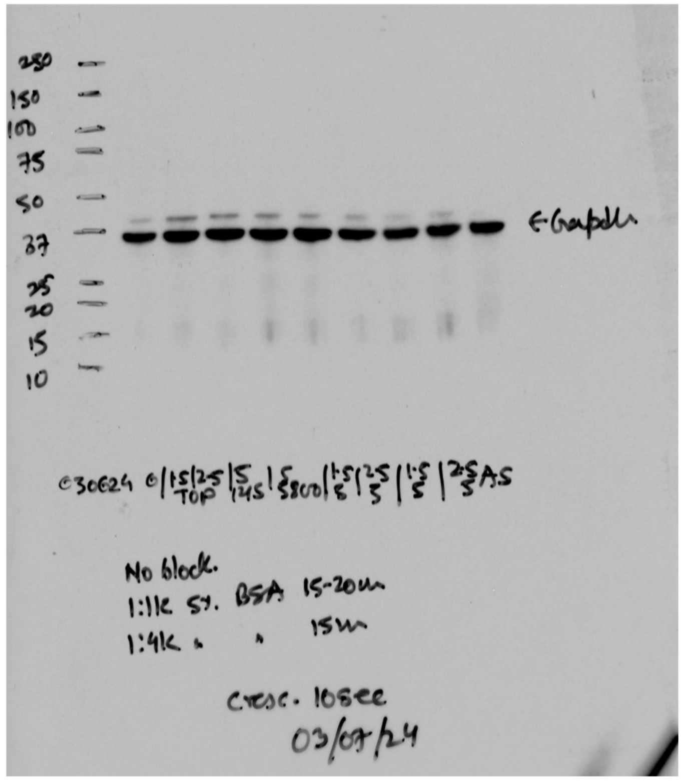

γH2AX

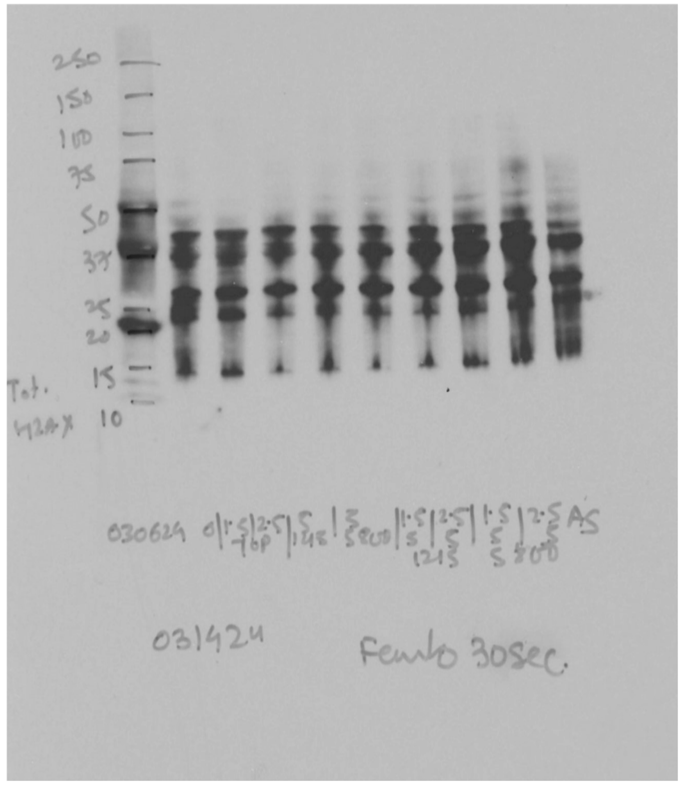

Total H2AX

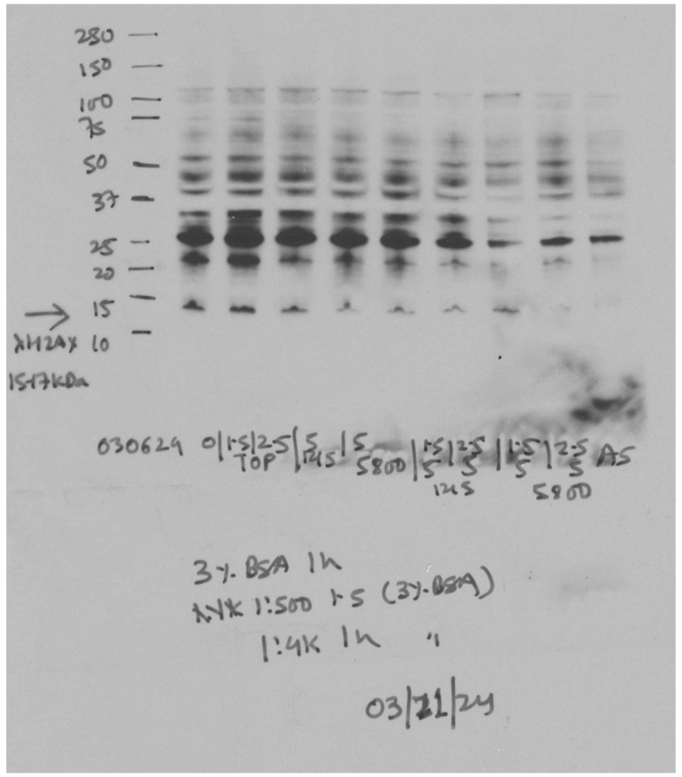

GAPDH

SK-N-AS

1C (middle panels)

**Topotecan (nM)**	**0**	**1.5**	**2.5**	**0**	**1.5**	**2.5**
**ATUX-1215 (µM)**	**0**	**0**	**0**	**5**	**5**	**5**

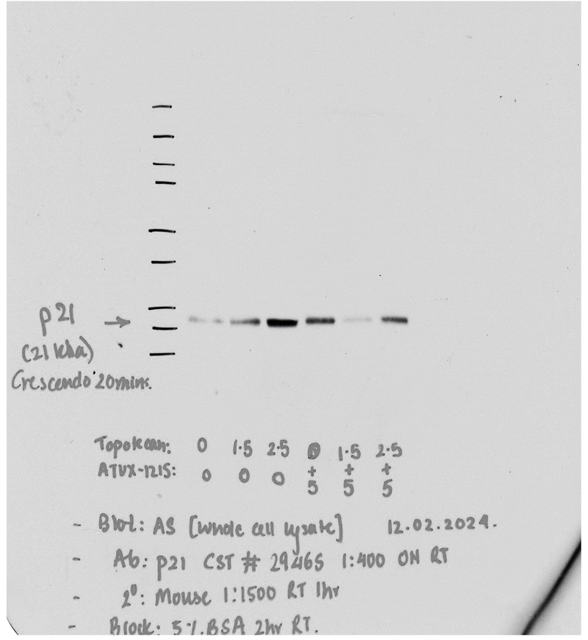

p21

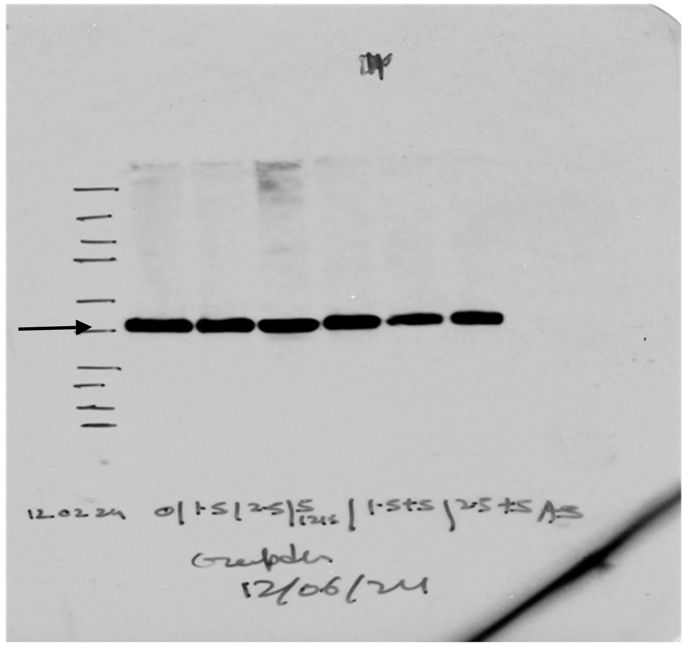

GAPDH

SK-N-AS

1C (lower panels)

**Topotecan (nM)**	**0**	**1.5**	**2.5**	**0**	**1.5**	**2.5**
**ATUX-1215 (µM)**	**0**	**0**	**0**	**5**	**5**	**5**

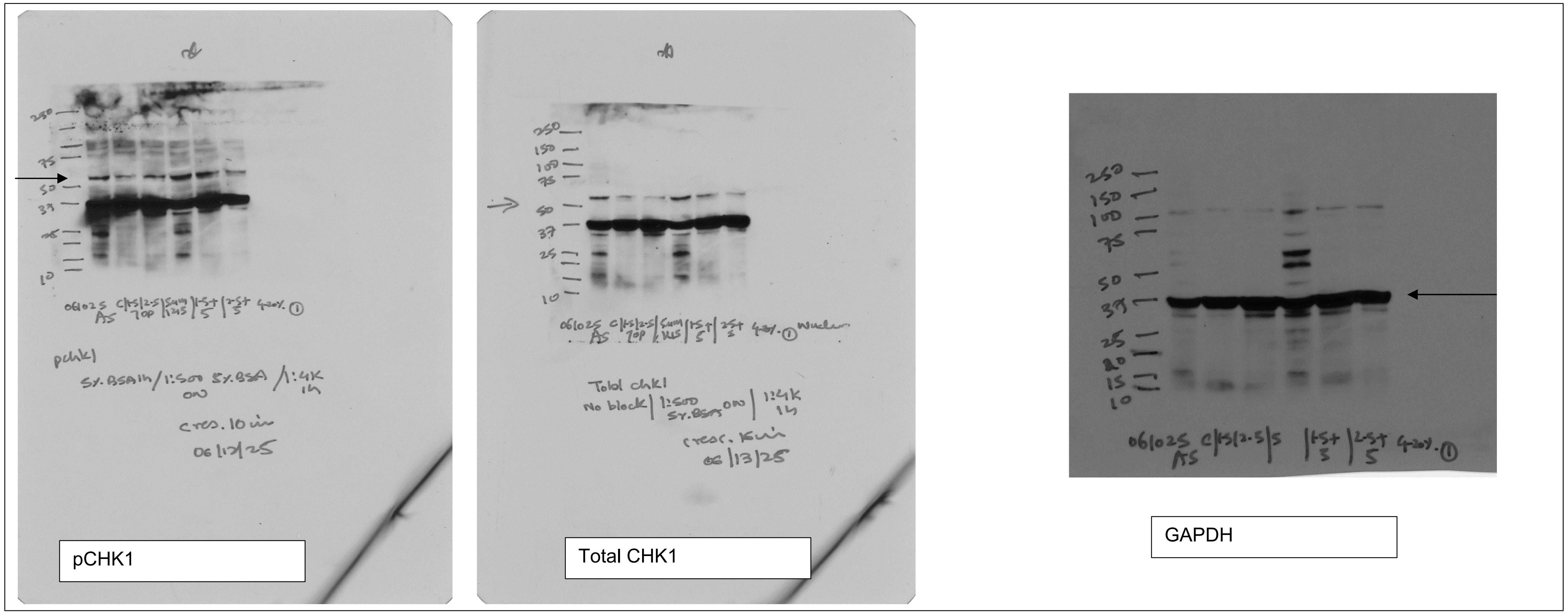

SK-N-BE(2)

1F (upper panels)

Topotecan (nM)	0	1	0	1
ATUX-1215 (μM)	0	0	5	5

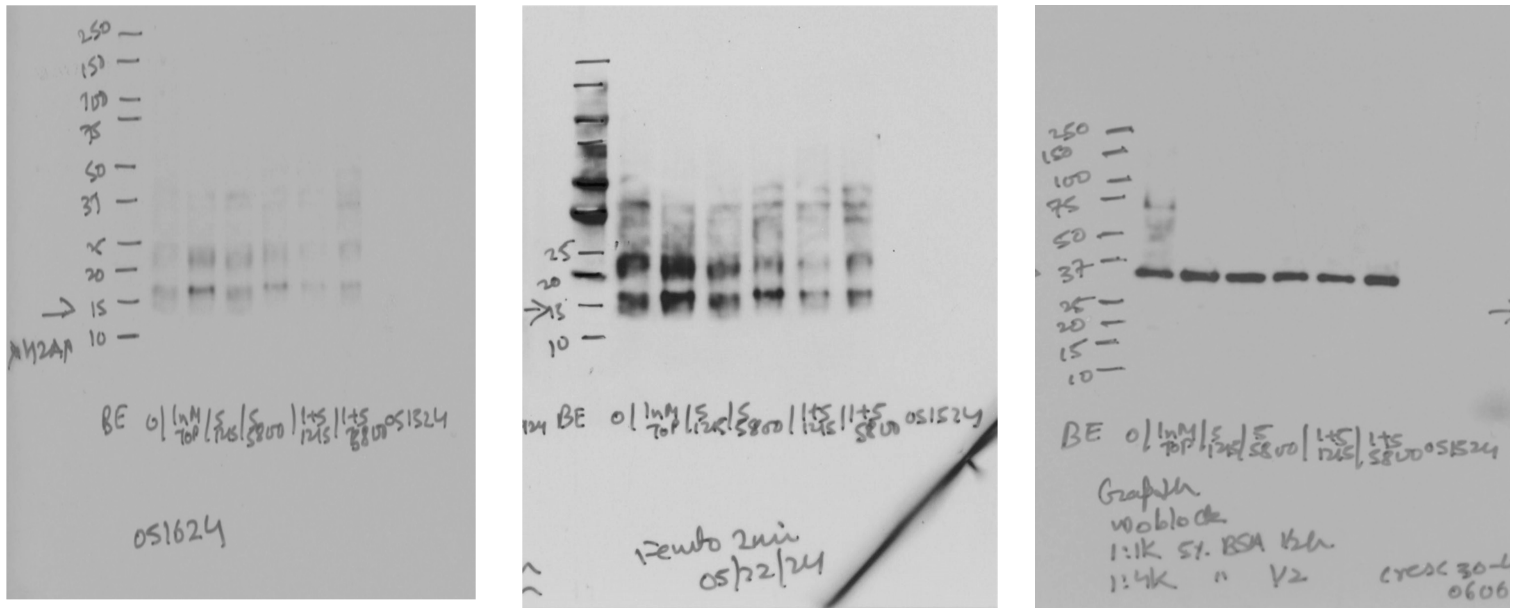

γ H2AX　　Total H2AX　　GAPDH

SK-N-BE(2)

1F (middle panels)

Topotecan (nM)	0	1	0	1
ATUX-1215 (μM)	0	0	5	5

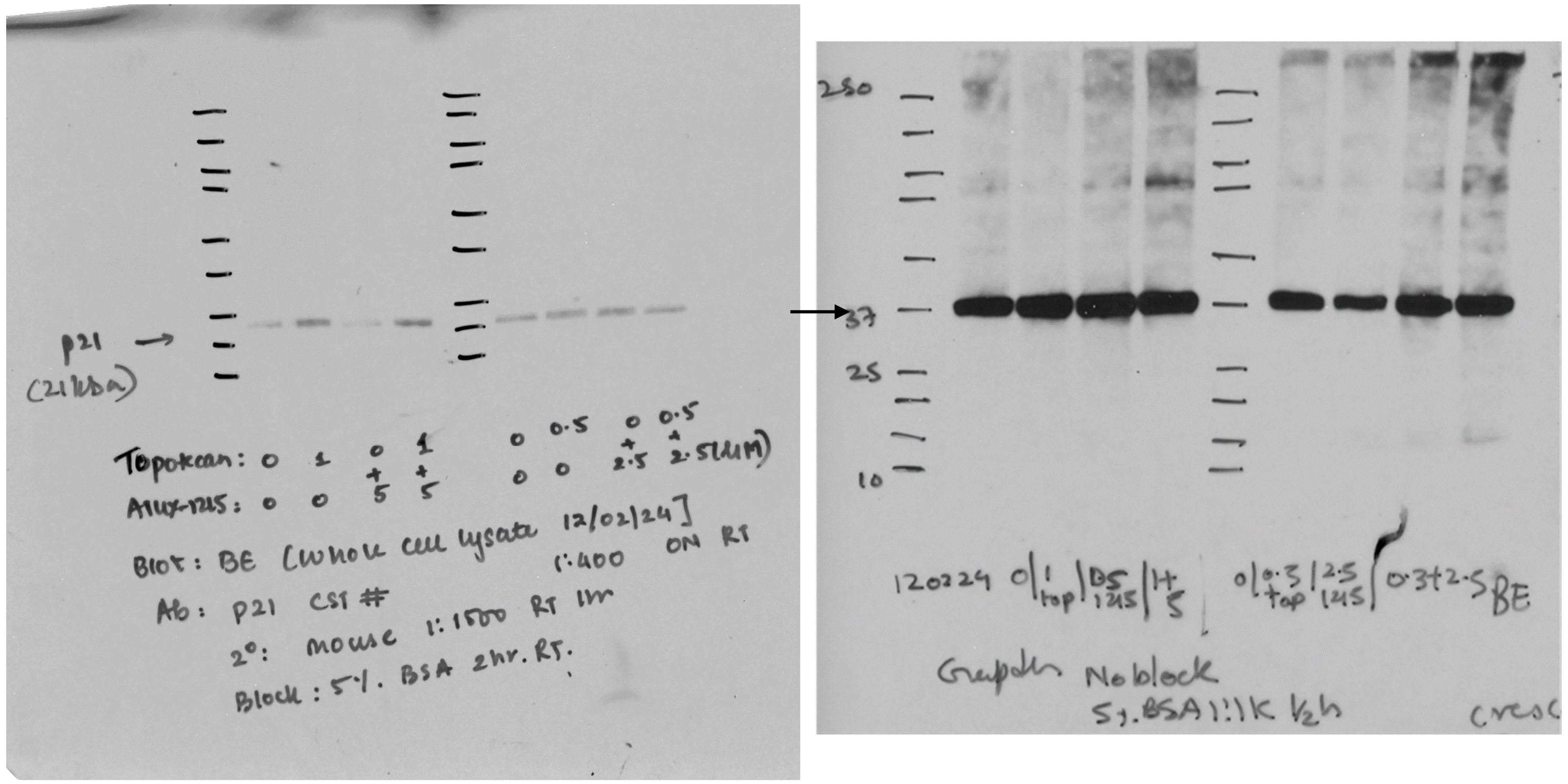

p21

GAPDH

SK-N-BE(2)

1F (lower panels)

Topotecan (nM)	0	1	0	1
ATUX-1215 (μM)	0	0	5	5

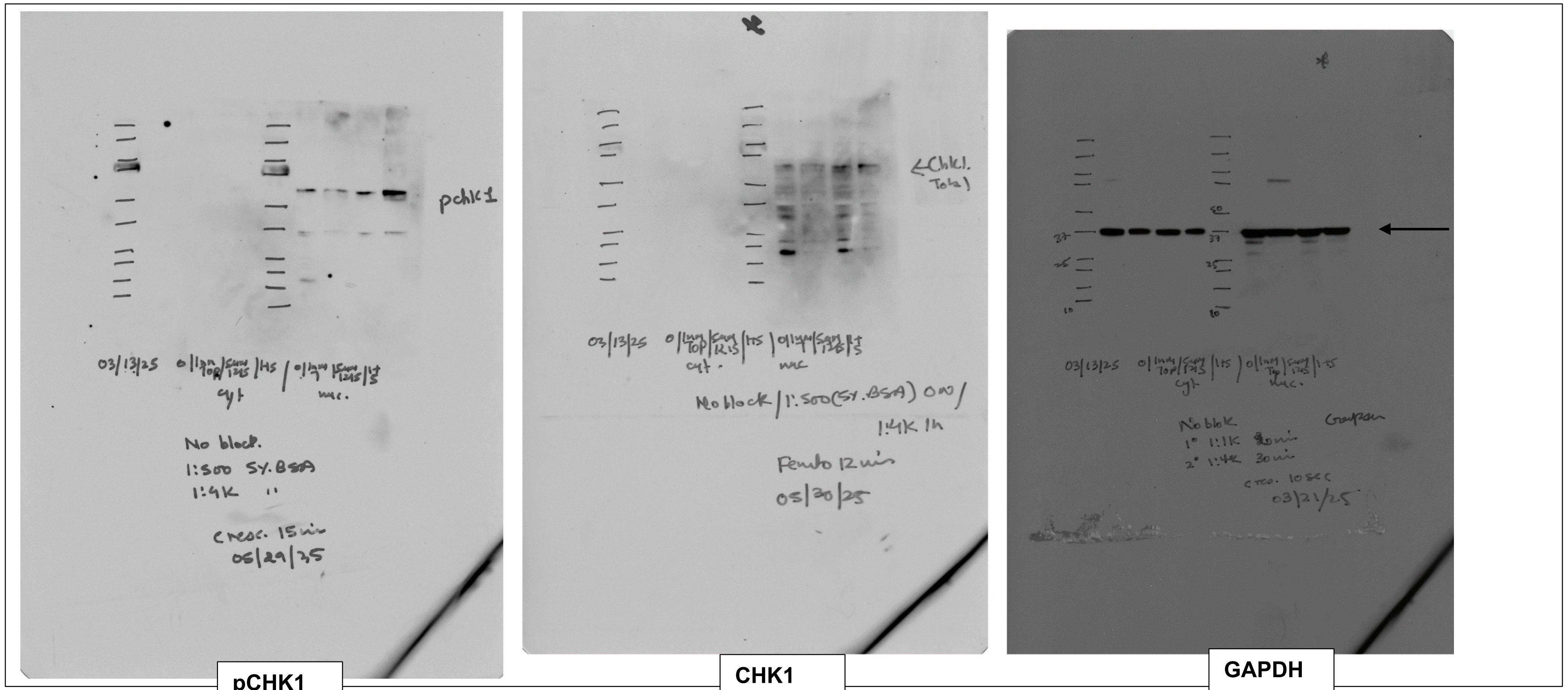

pCHK1 CHK1 GAPDH

SK-N-AS

Topotecan (nM)	0	1.5	2.5	--	1.5	2.5
ATUX- 1215 (μM)	0	--	--	5	5	5

4A

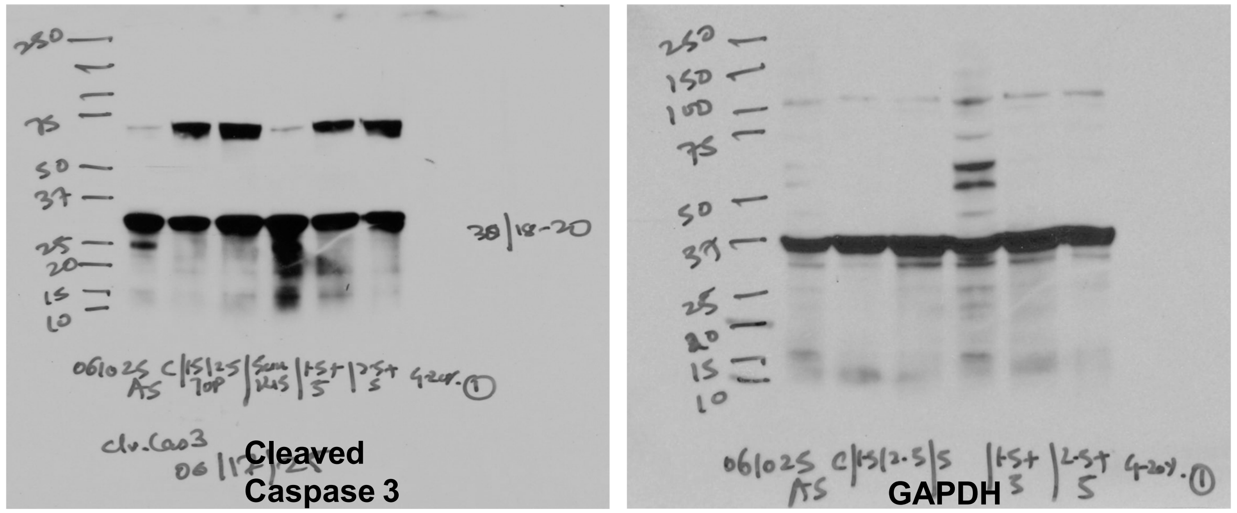

4B

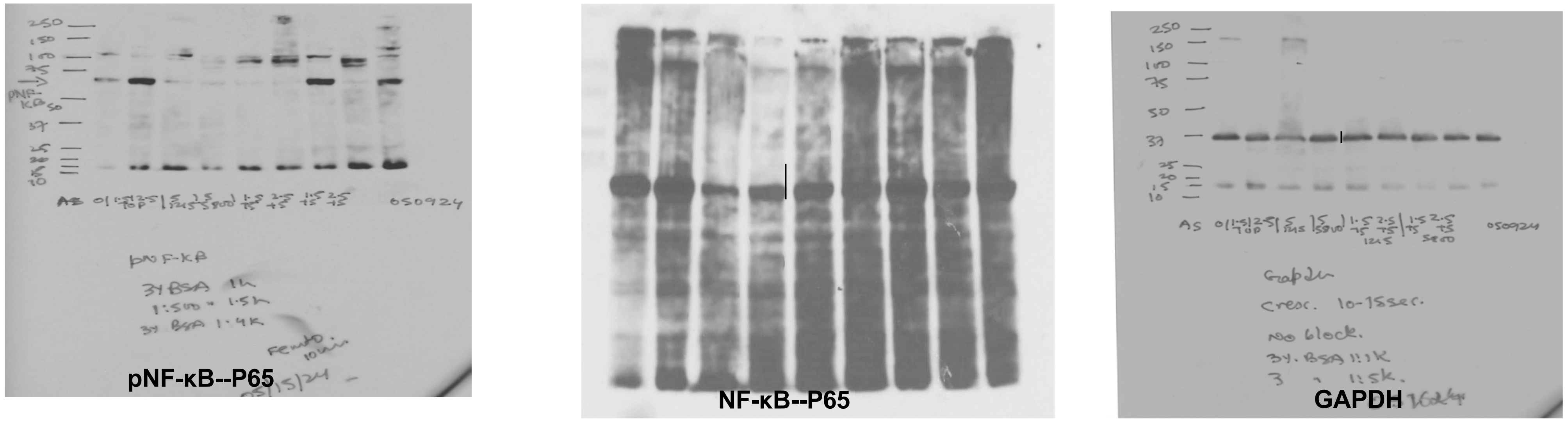

4C

SK-N-AS

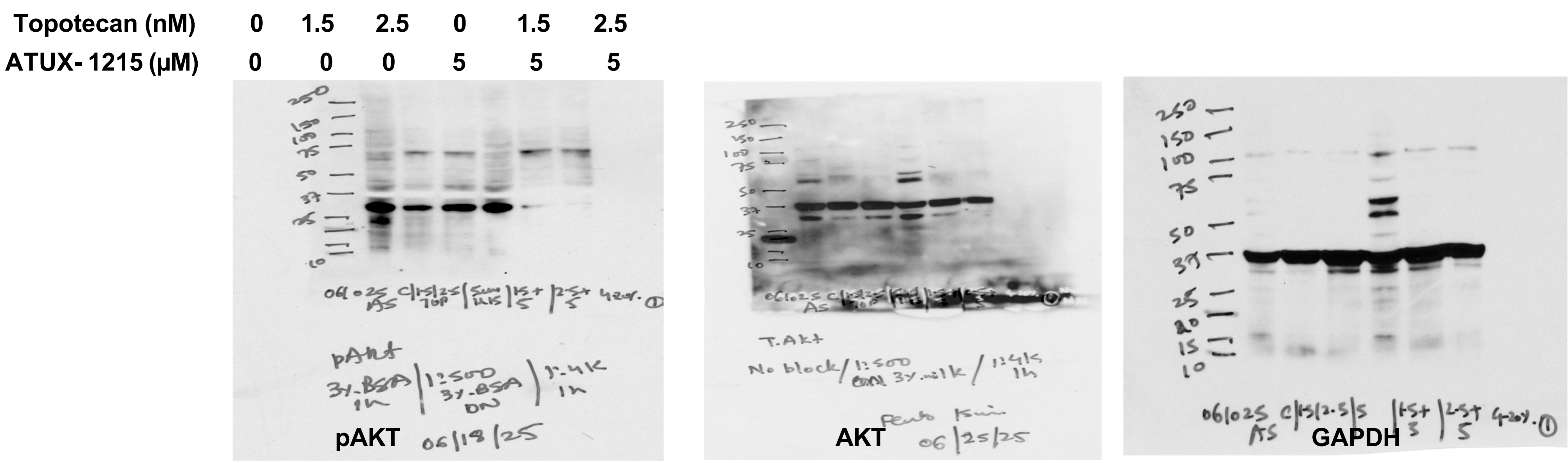

4D

SK-N-BE(2)

**Topotecan (nM)**	**0**	**1**	**0**	**1**
**ATUX- 1215 (μM)**	**0**	**0**	**5**	**5**

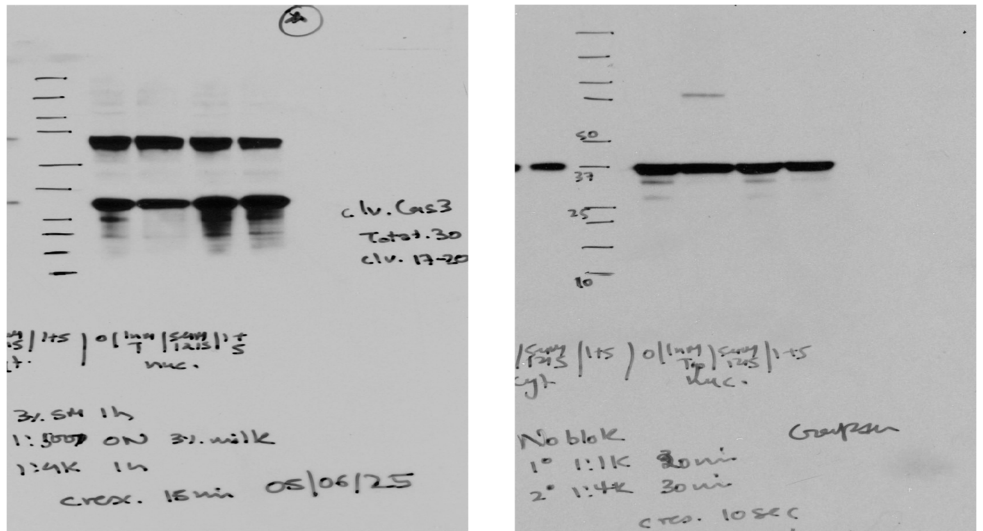

Cleaved Caspase 3 **GAPDH**

4E

SK-N-BE(2)

Topotecan (nM)	0	1	0	1
ATUX- 1215 (μM)	0	0	5	5

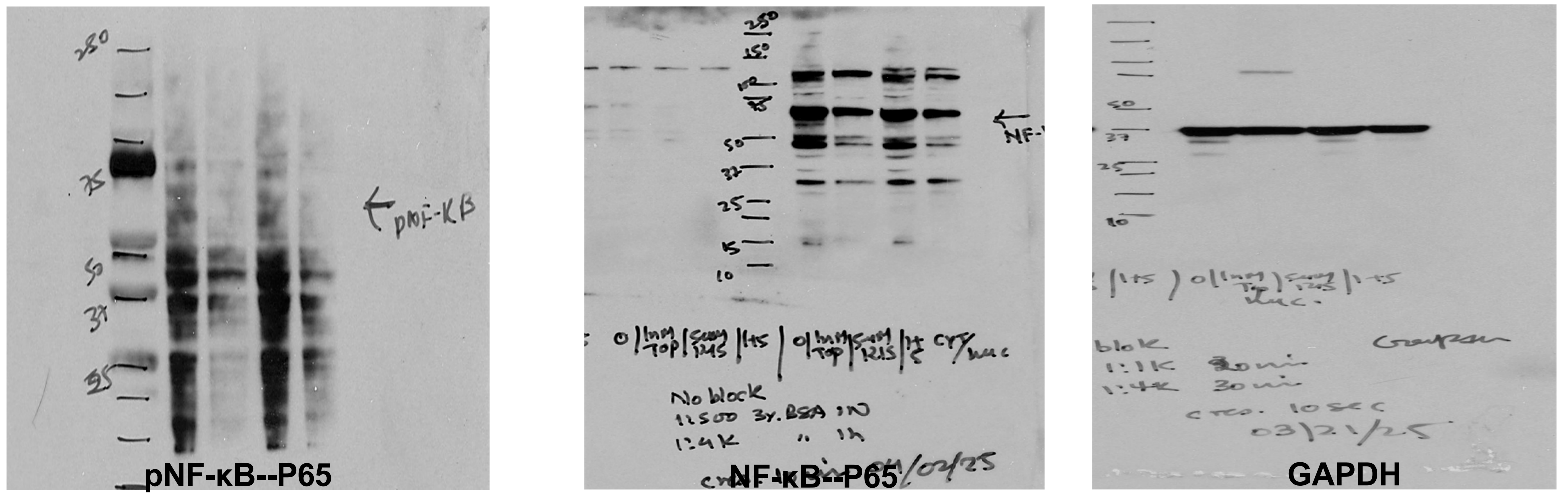

4F

Topotecan (nM)	0	1	0	1
ATUX- 1215 (μM)	0	0	5	5

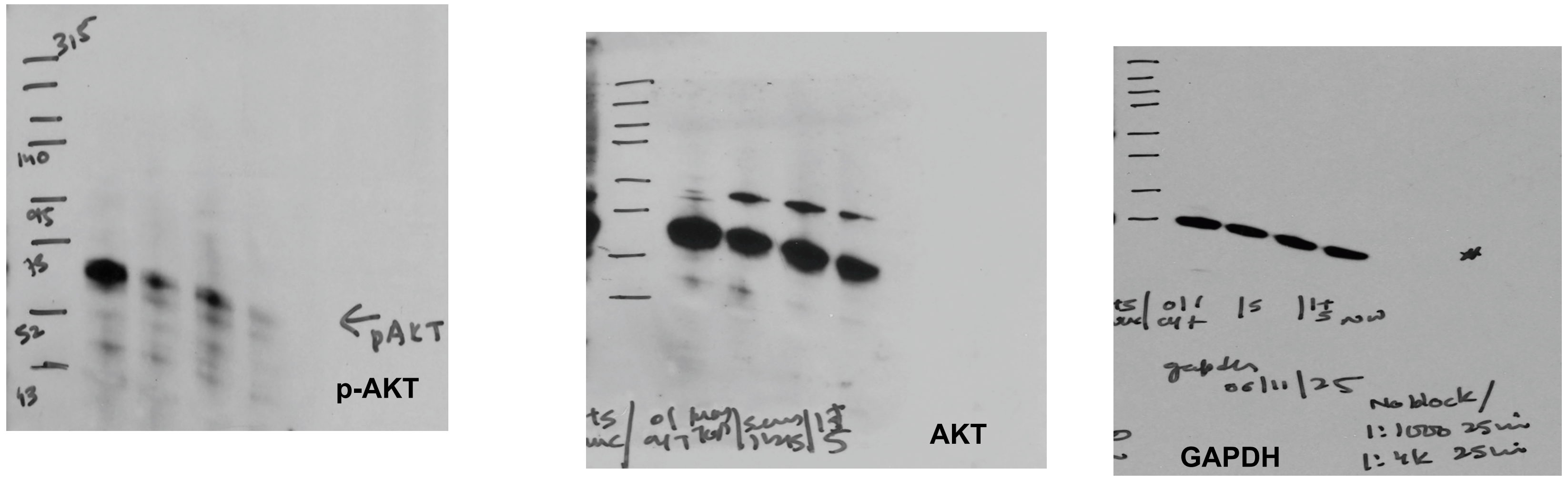

5D top panels

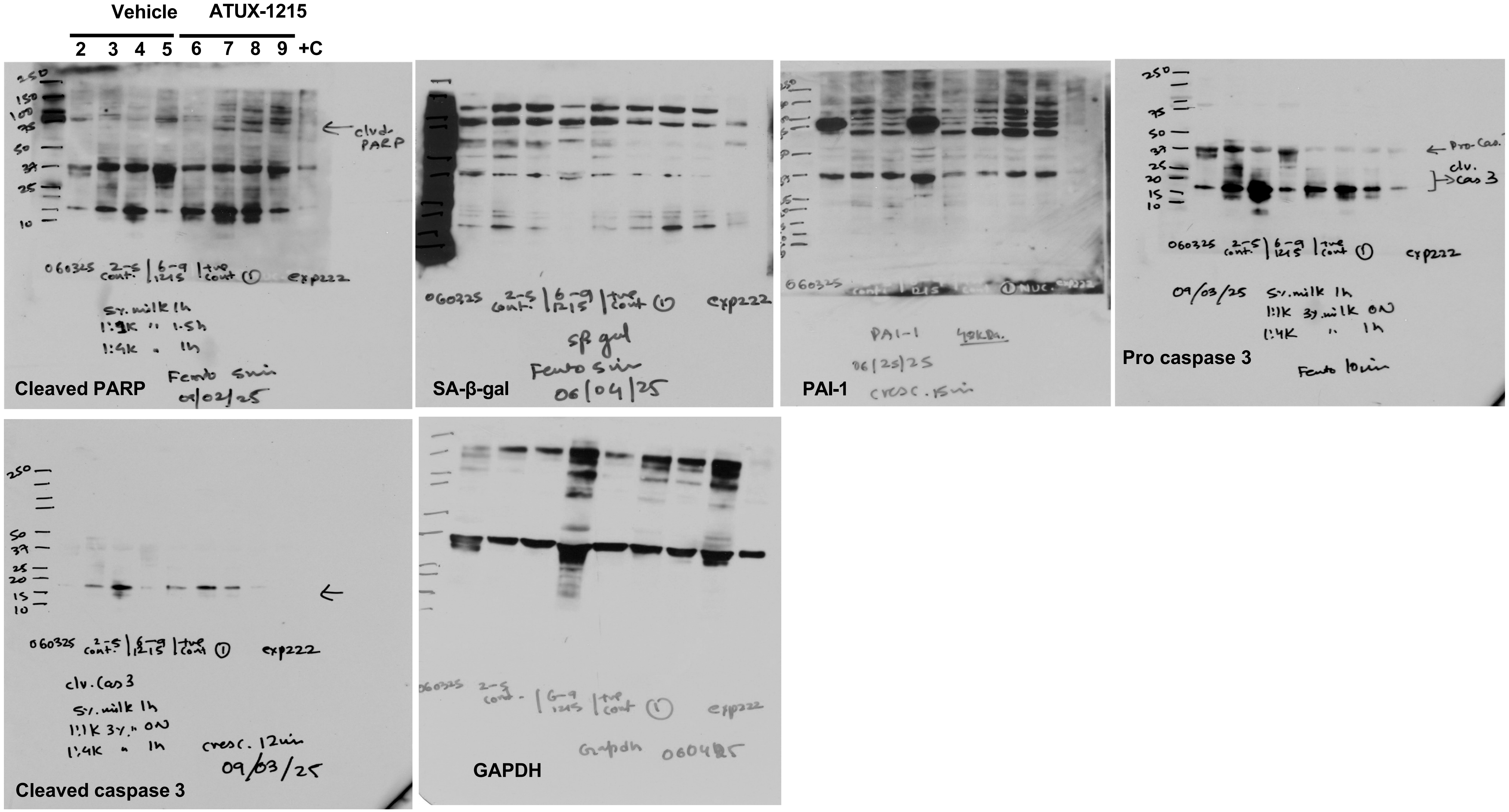

5D middle panels

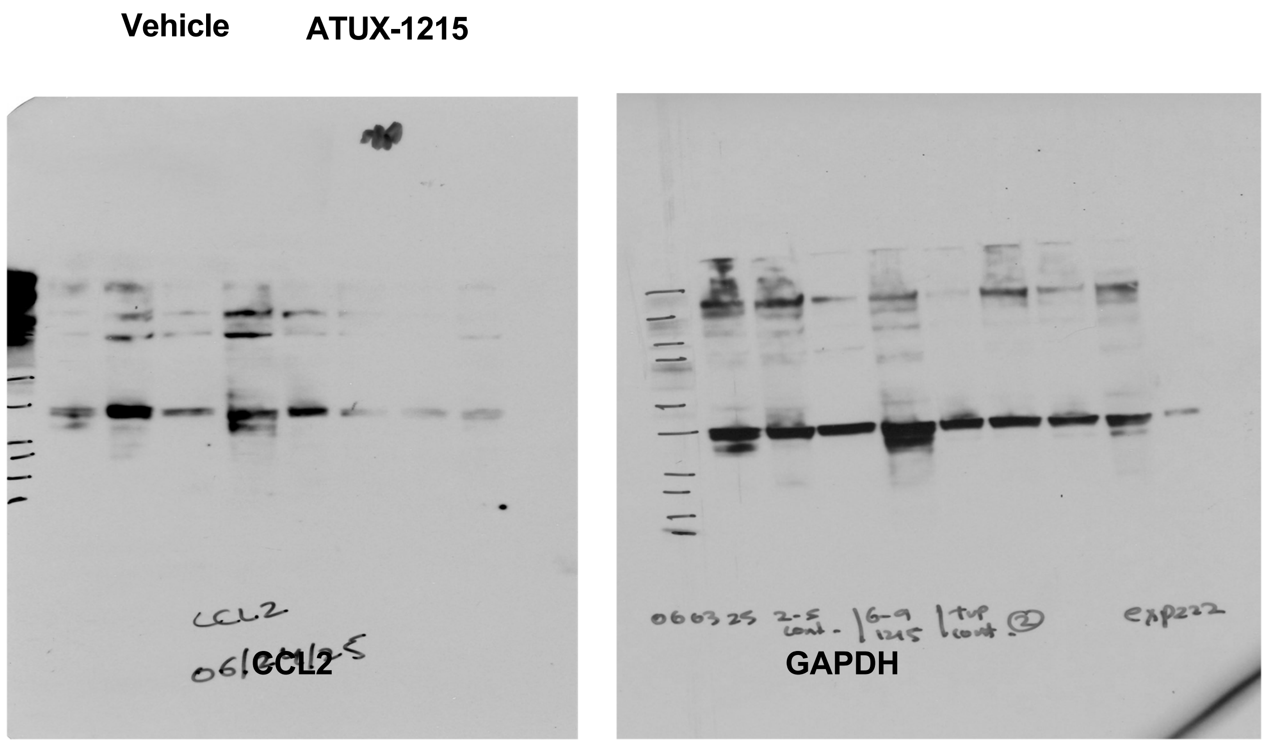

5D lower panel

Vehicle **ATUX-1215**

1 2 3 4 1 2 3 4

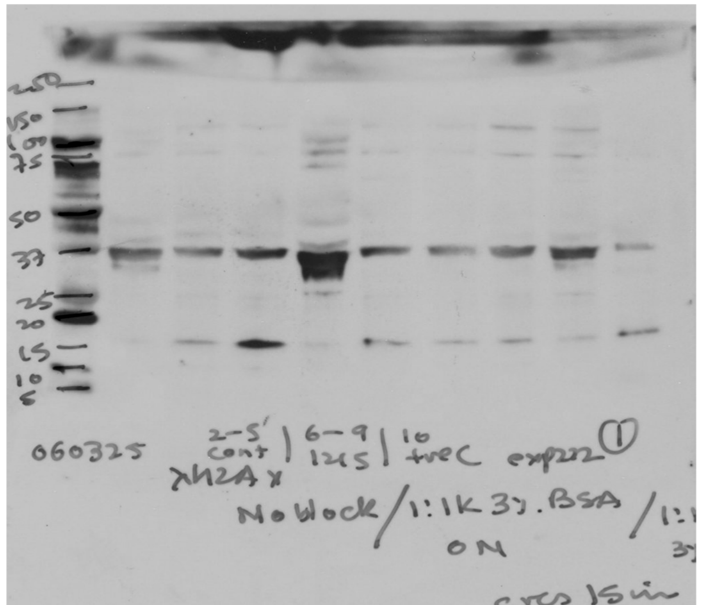

γH2AX

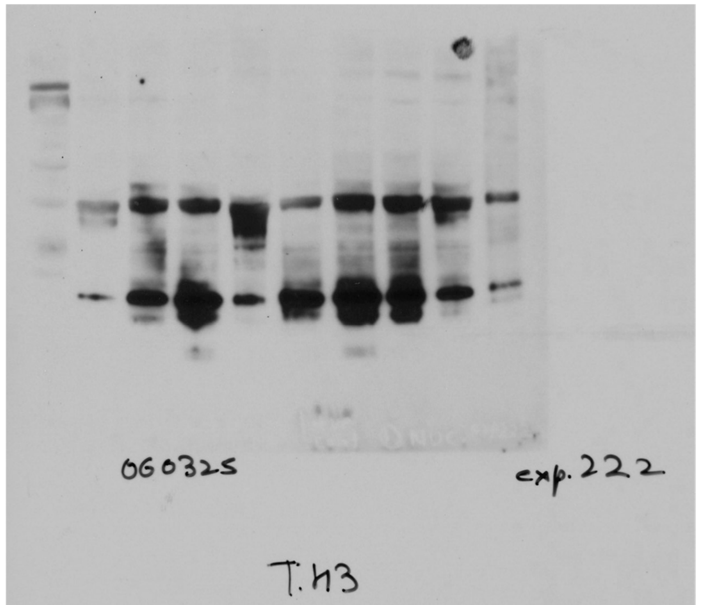

Total H3

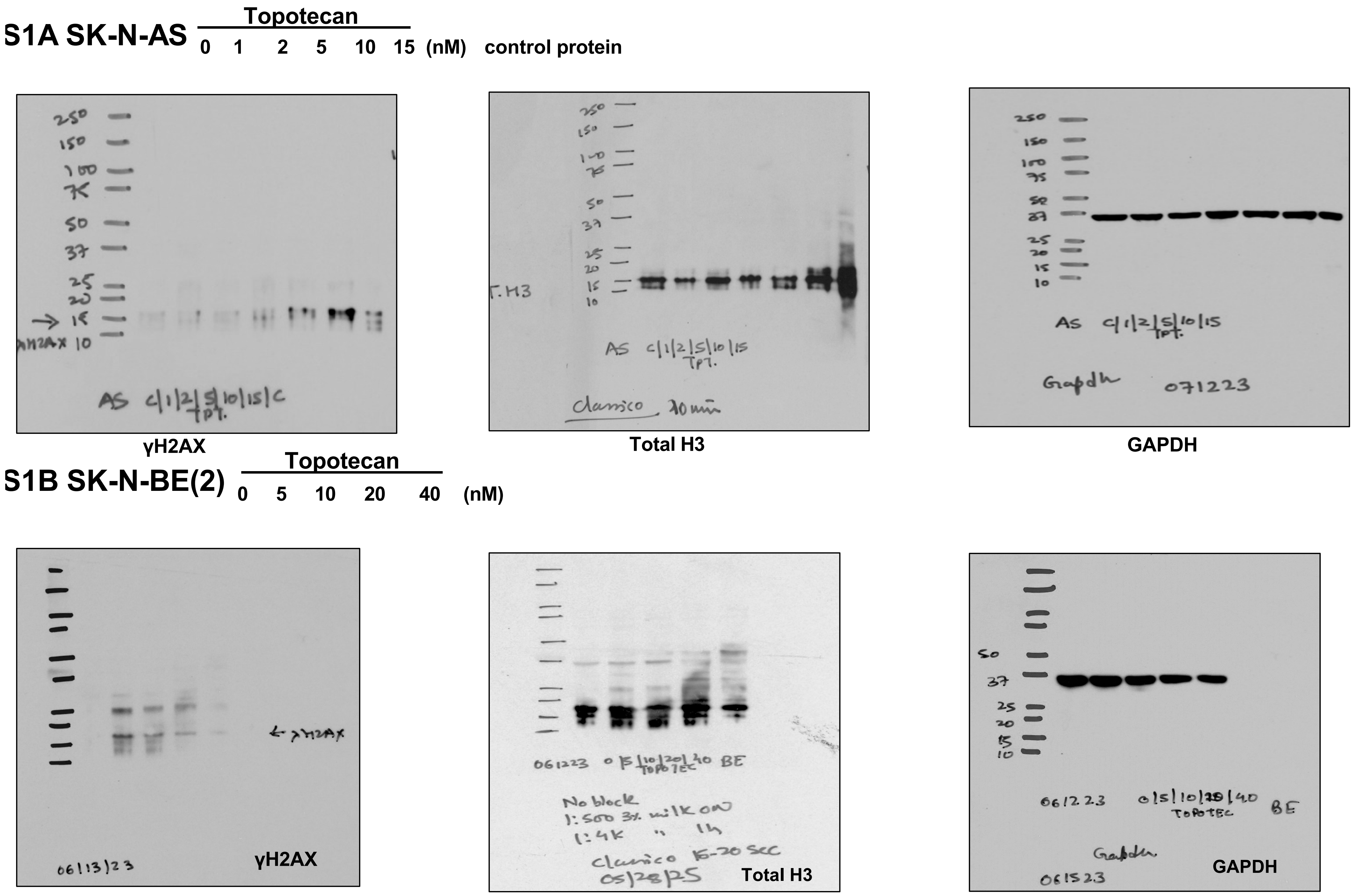
S1A SK-N-AS
Topotecan
0 1 2 5 10 15 (nM) control protein
γH2AX
Total H3
GAPDH
S1B SK-N-BE(2)
Topotecan
0 5 10 20 40 (nM)
γH2AX
Total H3
GAPDH

S4

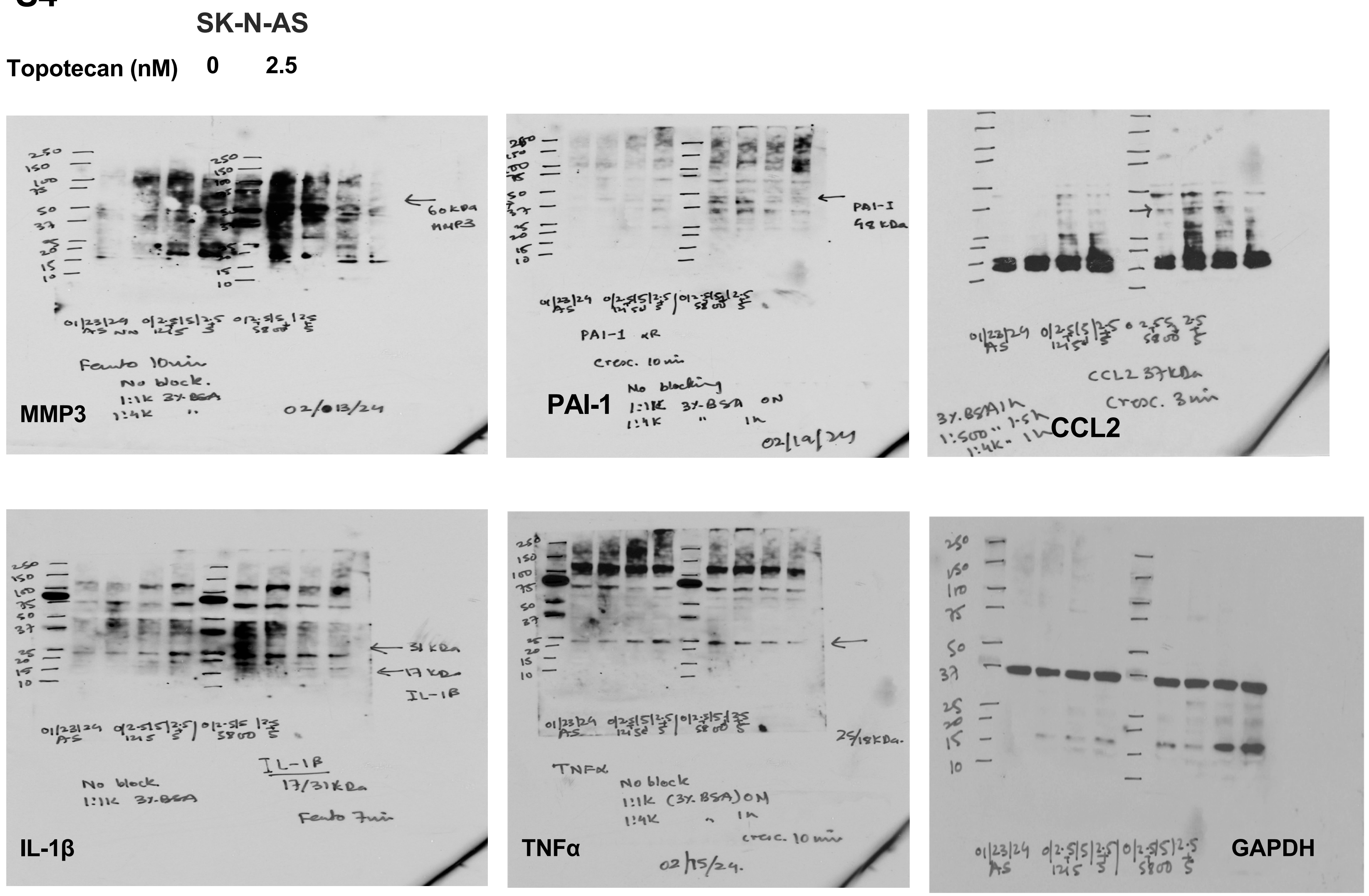

Supplement: Supplementary file 1 [file cancers-18-03088-s001.zip › cancers-4530007-supplementary.pdf]
